# Neutralizing IL-38 activates γδ T cell-dependent antitumor immunity and sensitizes for chemotherapy

**DOI:** 10.1136/jitc-2023-008641

**Published:** 2024-08-28

**Authors:** Priscila da Silva, Javier Mora, Xin You, Svenja Wiechmann, Mateusz Putyrski, Javier Garcia-Pardo, Aimo Kannt, Andreas Ernst, Bernhard Bruene, Andreas Weigert

**Affiliations:** 1 Faculty of Medicine, Institute of Biochemistry I, Goethe-University Frankfurt, Frankfurt, Germany; 2 Faculty of Microbiology, University of Costa Rica, San José, Costa Rica; 3 Centro de Investigación en Cirugía y Cancer (CICICA), University of Costa Rica, 2060 San José, Costa Rica; 4 Centro de Investigación en Enfermedades Tropicales (CIET), University of Costa Rica, San José, Costa Rica; 5 Fraunhofer Institute for Translational Medicine and Pharmacology (ITMP), Frankfurt, Germany; 6 Institut de Biotecnologia i de Biomedicina (IBB) and Departament de Bioquímica i Biologia Molecular, Universitat Autònoma de Barcelona, Barcelona, Spain; 7 Faculty of Medicine, Institute of Clinical Pharmacology, Goethe-University Frankfurt, Frankfurt, Germany; 8 Faculty of Medicine, Institute of Biochemistry II, Goethe-University Frankfurt, Frankfurt, Germany; 9 Partner Site Frankfurt, German Cancer Consortium (DKTK), Heidelberg, Germany; 10 Frankfurt Cancer Institute, Goethe-University Frankfurt, Frankfurt, Germany

**Keywords:** Breast Cancer, T cell, Chemotherapy, Antibody

## Abstract

**Background:**

The interleukin (IL)-1-family receptor antagonist IL-38 has emerged as a negative regulator of auto-inflammation. Given the intricate interplay between antitumor immunity and auto-inflammation, we hypothesized that blocking IL-38 may enhance tumor immune control.

**Methods:**

Our hypothesis was tested in the transgenic polyoma virus middle T oncoprotein mammary carcinoma model that is suitable for identifying strong immunomodulators. To investigate the effect of acute IL-38 blockade, we used a neutralizing antibody, alone or in combination with chemotherapy. Immune cell composition and location in tumors were determined by flow cytometry and immunohistochemistry, respectively. The role of γδ T cells was studied using an antibody blocking γδ T-cell receptor signaling. Whole transcriptome RNA sequencing and RNA expression analysis were employed to determine mechanisms downstream of IL-38 neutralization. Additionally, in vitro assays with γδ T cells, CD8+ T cells and cDC1, followed by in vivo CD8+ T cell depletion, were performed to study the underlying mechanistic pathways.

**Results:**

Both, genetic ablation of IL-38 and neutralization with the antibody, reduced tumorigenesis, and IL-38 blockade improved chemotherapy efficacy. This was accompanied by an augmented lymphocyte infiltrate dominated by γδ T cells and CD8+ T cells, and signaling through the γδ-T-cell receptor was required for CD8+ T cell infiltration. Rather than directly interacting with CD8+ T cells, γδ T cells recruited conventional dendritic cells (cDC1) into tumors via the chemokine Xcl1. cDC1 in turn activated CD8+ T cells via the Notch pathway. Moreover, IL-38 negatively correlated with cDC1, XCL1-producing γδ T cells, T-cell infiltrates and survival in patients with mammary carcinoma.

**Conclusions:**

These data suggest that interfering with IL-38 improves antitumor immunity even in immunologically cold tumors.

**WHAT IS ALREADY KNOWN ON THIS TOPIC**

- Interleukin (IL)-38 is a cytokine that is produced by dying cells and limits inflammatory reactions. Dying tumor cells can suppress protective immunity in tumors, but the role of IL-38 in this context was unknown.

**WHAT THIS STUDY ADDS**

- This study for the first time combines genetic and pharmacological approaches to elucidate the impact of IL-38 on antitumor immunity in mammary cancer. It suggests that IL-38 acts on tumor-infiltrating γδ T cells to avoid an effective cytotoxic antitumor immune response.

**HOW THIS STUDY MIGHT AFFECT RESEARCH, PRACTICE OR POLICY**

- This study suggests that interfering with IL-38 may increase antitumor immune responses, particularly under conditions when tumor cell death is induced, for example, following chemotherapy.

## Introduction

 Interleukin-1 (IL-1) family cytokines coordinate innate and adaptive immune responses.^1^ They are roughly divided into receptor agonists and antagonists having either pro-inflammatory or anti-inflammatory properties.^2 3^ IL-38 is a recently described member of the IL-1 family that shows 41% and 43% amino acid identity with the receptor antagonists IL-1RA and IL-36RA, respectively.^4^ Accordingly, IL-38 was shown to suppress inflammatory reactions in human immune cells and various mouse models.^5 6^ Particularly, IL-38 appears to play a role in limiting IL-17 production and IL-17-dependent chronic inflammatory reactions, with IL-17 concentrations being consistently increased in IL-38 deficient mice during inflammation.^7^,^12^ Primary target cells of IL-38 include macrophages^11^,^13^ to limit the production of cytokines that drive TH17 responses, and IL-17-producing T cells. For example, IL-38 acts directly on dermal γδ T cells to limit IL-17 production in a model of skin inflammation.^10^

IL-17 cytokines are major driver of auto-inflammatory reactions.^14^ The side effects of immune checkpoint inhibitors clearly delineate a close relationship between antitumor immunity and auto-inflammatory reactions,^15^ indicating that IL-17, and in consequence, IL-38, may be involved in antitumor immunity. Although the relationship between IL-17 and tumor progression appears to be complex,^16^ we wondered if targeting IL-38 might affect tumor immune control. Previously, high expression of IL-38 in patience with lung adenocarcinoma was associated with tumor progression and poor survival, and correlated positively with programmed death-ligand 1 (PD-L1) expression.^17^ IL-38, when being overexpressed in Lewis lung carcinoma cells, favored tumor growth, accompanied by decreased CD8+ T cell infiltration.^18^ However, mechanisms and the role of endogenous IL-38 in cancer progression remained elusive.

## Methods

### Animal experiments

The C57BL/6N IL-38 knockout (KO) strain was previously described.^10^ For the polyoma virus middle T oncoprotein (PyMT) model, IL-38 KO mice were crossed with the PyMT mammary carcinoma strain, previously bred into a C57BL/6 background.^19^ Only female PyMT mice were used. Tumor development in PyMT wildtype (WT) versus IL-38 KO mice was monitored once a week for up to 8 weeks, starting at 12 weeks using electronic calipers. For IL-38 neutralization, PyMT WT mice were intraperitoneally (i.p.) injected with 100 µg of either anti-IL-38 antibody (e04, in-house generated) or IgG1 (Human IgG1, Bio X Cell) isotype control. For CD8+ T cell depletion, PyMT WT mice were injected with 100 µg of anti-IL-38 antibody (e04) in combination with either 250 µg of anti-CD8 antibody (anti-mouse CD8α, Bio X Cell) or IgG2b (rat IgG2b, Bio X Cell) isotype control. In both experiments, the treatment started once the first tumor reached a size of 0.6 cm in diameter. The antibodies were injected once a week for 5 weeks. In the therapeutic model of chemoresistance, PyMT WT mice were i.p. injected with 5 mg/kg of doxorubicin (Teva Pharma) in combination with either IgG1 isotype control or anti-IL-38 antibody. The mice were treated with five once-weekly cycles of chemotherapy once the first tumor reached a size of 1.0 cm in diameter. For γδ T-cell receptor (TCR) neutralization, mice were separated into four groups, in which PyMT WT and IL-38 KO mice were treated with either anti-γδ TCR antibody (UC7-13D5, Bio X Cell) or IgG (polyclonal Armenian hamster, Bio X Cell) isotype control once a week for 5 weeks. Mice were i.p. injected once with 500 µg of antibodies, followed by 4 weekly injections of 200 µg.^20^ The treatment started at week 13 and tumor growth was monitored once a week. Group sizes were determined based on prior experience with the model. No exclusion criteria were defined and no animals were excluded. Due to the nature of the model (treatment start for every single animal depends on individual parameters), simple randomization (no confounder minimization) was done and the experimenters were not blinded. Data analysis was done in a blinded manner. Animals were housed in groups at the Zentrale Forschungseinrichtung (animal testing facility), Faculty of Medicine, Goethe University Frankfurt. Humane endpoints were individual tumor size >1.5 cm diameter, cumulative tumor size >3 cm diameter, body condition score, and weight loss >20%.

### Flow cytometry

For preparing single-cell suspensions, PyMT tumors were processed with the Tumor Dissociation Kit (Miltenyi Biotec) and gentleMACS Dissociator (Miltenyi Biotec) according to standard protocols. The samples were filtered using 70 µm cell strainers (BD Biosciences), blocked with 2% Fc receptor binding inhibitor (Miltenyi Biotec) and incubated with fluorochrome-coupled antibodies (online supplemental table S2). For intracellular cytokine staining, single-cell suspensions were incubated with 5 µg/mL brefeldin A (eBioscience), Golgi stop and PMA/ionomicyn (BD Biosciences) for 4 hours at 37°C followed by cell surface marker staining. Then, cells were fixed, permeabilized (Cytofix/Cytoperm Fixation/Permeabilization Kit, BD Biosciences), and stained with anti-interferon (IFN)-γ and anti-IL-17 antibodies. Conventional dendritic cells (cDC1) from PyMT tumors, CD8+ T cells and γδ T cells from spleen of C57BL/6N mice were isolated using an FACSymphony S6 cell sorter (BD Bioscience). Samples were acquired with an FACSymphony A5 or FACSymphony S6 flow cytometer (BD Bioscience) and the data was analyzed in FlowJo software V.10 (Tree Star). All primary antibodies and secondary reagents were titrated to determine the optimal concentration. Comp-Beads (BD Bioscience) were used for single-color compensation to create multicolor compensation matrices. For the gating strategy, fluorescence minus one controls were applied. The instrument was controlled daily by calibrations with Cytometer Setup and Tracking beads (BD Bioscience).

### Quantitative real-time PCR

Epithelial cells, endothelial cells, dendritic cells (DCs), CD8+ T cells and γδ T cells were isolated from PyMT tumors treated in vivo with either anti-IL-38 antibody or IgG isotype control followed by RNA extraction with Absolutely RNA Microprep Kit (Agilent) and reverse transcription reaction by Sensiscript RT Kit (Qiagen). Total RNA was extracted using TRIzol Reagent (Life Technologies). For the transcription reaction Maxima cDNA Synthesis Kit (Thermo Fisher Scientific) was used. Quantitative real-time PCR (qPCR) was performed with PowerUp SYBR Green Master Mix (Thermo Fisher Scientific) and QuantStudio 5-Real-Time PCR (Thermo Fisher Scientific). Relative messenger RNA (mRNA) expression was calculated by ΔΔCt method and normalized to *Rps27a* housekeeping gene. The following murine primers were used:

Dll1 F: 5'-AGATAACCCTGACGGAGGCT-3', R: 5'-ACACACTTGGCACCGTTAGA-3'

Dll4 F: 5'-CAGTTGCCCTTCAATTTCACCT-3', R: 5'-AGCCTTGGATGATGATTTGGC-3'

Jag1 F: 5'-CCTCGGGTCAGTTTGAGCTG-3', R: 5'-CCTTGAGGCACACTTTGAAGTA-3'

Hes1 F: 5'-ACACCGGACAAACCAAAGAC-3', R: 5'-ATGCCGGGAGCTATCTTTCT-3'

HeyL F: 5'-GAATTGCGACGATTGGTCCC-3', R: 5'-TCTTCAAGTGATCCACGGTCAT-3'

Notch1 F: 5'-GATGGCCTCAATGGGTACAAG-3', R: 5'-TCGTTGTTGTTGATGTCACAGT-3'

Notch2 F: 5'-CTGTGAGCGGAATATCGACGA-3', R: 5'-ATAGCCTCCGTTTCGGTTGG-3'

Notch3 F: 5'-AGTGCCGATCTGGTACAACTT-3', R: 5'-CACTACGGGGTTCTCACACA-3'

Notch4 F: 5'-GAACGCGACATCAACGAGTG-3', R: 5'-GGAACCCAAGGTGTTATGGCA-3'

Xcl1 F: 5'-ACGAAATGCGAAATCATGTGC-3', R: 5'-CTGTGTCGTCTCCAGGACAA-3'

IIf10 F: 5'-AGAGTGAACCCTCCACCCAT-3', R: 5'- AAGATCTCAGACTGGGGGCA-3'

IL36R F: 5'-GAAACAAACGGGGCAGTAAATC-3', R: 5'-GGTGAACTCTAAGGTGTCTGTTG-3'

IL1RAPL1: Mm Il1rapl1 QT00292691 (Qiagen).

### Selection and production of Fabs binding to mouse IL-38

A synthetic Fab-phage library was used to generate binders to murine IL-38 (online supplemental methods). We followed the standard phage display workflow to identify positive Fabs.^21^ Briefly, after five rounds of selection, 96 individual clones were assessed by phage ELISA for binding to mIL-38. DNA of positive clones was sequenced. Plasmids of individual clones were transformed into BL21 *Escherichia coli*. Cultures were grown in LB-media at 30°C until an OD600 of 0.5 was reached. Antibody Fab expression was induced with 1 mM IPTG, followed by overnight incubation at 30°C with shaking at 250 rpm. Cells were harvested by centrifugation and lysed according to.^21^ Fabs were purified using HiTrap Protein G (Cytiva) and gravity flow. Eluted Fabs were dialyzed into 1× phosphate-buffered saline (PBS) and analyzed using sodium dodecyl sulfate polyacrylamide gel electrophoresis (SDS-PAGE).

### Production of IgG e04

Fab e04 DNA was converted into full-length IgG1 format and cloned into mammalian expression plasmid pcDNA3.4 (Thermo Fisher). Expi293F cells (Thermo Fisher) were cultured and transfected following the manufacturer’s protocol. Six days post-transfection, supernatant was collected and e04 IgG was purified using HiTrap Protein G (Cytiva) and gravity flow. Eluted IgG was dialyzed into 1× PBS and analyzed using SDS-PAGE.

### Bio-layer interferometry

Kinetic binding assays were performed on an Octet RED96 instrument (Sartorius). Proteins were supplemented with 0.1% bovine serum albumin (BSA) and 0.02% Tween 20. mIL-38-Avi was immobilized on Streptavidin (SA) Biosensors (Sartorius) at a concentration of 2 µg/mL. The association of Fabs or IgG was analyzed at concentrations starting from 250 to 7.8 nm in 1:1 dilution steps. Dissociation was measured in dialysis buffer (150 mm NaCl, 50 mm Tris-HCl, pH 7.5) supplemented with 0.1% BSA and 0.02% Tween 20. A 1:1 analyte model with Global Fit was used for affinity calculations of Fabs and 1:2 bivalent model for IgG e04.

### T-cell co-culture

γδ T cells and CD8+ T cells were isolated from murine spleens by fluorescence-activated cell sorting (FACS) and co-cultured at a ratio of 1:10. The cells were cultured in T-cell medium Roswell Park Memorial Institute (RPMI) 1640 with 5 mM glutamine, 100 U/mL penicillin, 100 µg/mL streptomycin, 10% heat-inactivated fetal calf serum (FCS), 1% non-essential and essential amino acids, 1% sodium pyruvate and 1% 4-(2-hydroxyethyl)-1-piperazineethanesulfonic acid (HEPES) followed by activation with mouse T-cell activator CD3/CD28 Dynabeads (Thermo Fisher Scientific), 10 ng/mL of recombinant murine (rm) IL-23 and rm IL-1β (both from Bio-Techne). Cells were supplemented with 10 ng/mL rm IL-2 (PrepoTech) at days 0, 2 and 5 and daily with 100 ng/mL rm IL-38 (AdipoGen) and 50 μM β-mercaptoethanol. Cells were cultured for up to 5 days. At the endpoint, supernatants were collected for cytokine determination and cell proliferation was analyzed by flow cytometry.

### CD8+ T-cell proliferation assay

CD8+ T cells from murine spleen and cDC1 from PyMT tumors treated in vivo with anti-IL-38 antibodies were isolated by FACS-sorting. CD8+ T cells and cDC1 were co-cultured at a ratio of 1:40 in the T-cell medium. Cells were pre-incubated with either 100 nM anti-IL-38 or 100 nM IgG isotype control, activated with mouse T-cell activator CD3/CD28 Dynabeads (Thermo Fisher Scientific) and supplemented with 10 ng/mL rm IL-2 (PrepoTech) at days 0, 2 and 5 and 50 μM β-mercaptoethanol daily. For the Notch signaling blockade, 5 µM γ-secretase inhibitor DAPT (Abcam) was added daily. For CD8+ T cell culture, 100 ng/mL rm IL-38 (AdipoGen) was added daily. Supernatants were collected for cytokine determination at days 3, 5 and 7 and cell proliferation was analyzed by flow cytometry.

### cDC1 migration

γδ T cells isolated from murine spleen were cultured in T-cell medium, activated with mouse T-cell activator CD3/CD28 Dynabeads (Thermo Fisher Scientific), except for the negative control, and supplemented with 10 ng/mL rm IL-2 (PrepoTech), 100 ng/mL rm IL-38 (AdipoGen) and 100 mM β-mercaptoethanol. Supernatants were collected 24 hours after seeding for the determination of Xcl1 levels.

Boyden chamber assays were performed using a 96 well-plate with 4.26 mm transwell inserts with 5.0 µm pore polycarbonate membrane (Corning). Briefly, 1.4×10^4^ DC-enriched murine splenocytes (EasySep Mouse Pan-DC Enrichment Kit II, STEMCELL Technologies) were suspended in serum-free RPMI 1640 medium and added to transwell inserts. Supernatants collected after 24 hours of γδ T cells stimulation (described above) were pre-incubated with either 15 µg/mL IgG isotype control (R&D Systems) or 15 µg/mL anti-Xcl1 (R&D Systems) and added to the bottom compartment. Cells were harvested 2 hours later and migrated and non-migrated cells were analyzed by FACS. The percentage of migrated cDC1 was determined by the ratio of migrated/non-migrated cells.

### XCL1 ELISA

Xcl1 levels were determined from supernatants collected from γδ T-cell cultures using Mouse Xcl1/Lymphotactin DuoSet ELISA Kit (R&D Systems) according to the manufacturer’s instructions.

### Cytometric bead array

To measure the cytokines in PyMT tumors and cell culture supernatants, Cytometric Bead Array Flex Sets (BD Biosciences) were used for murine IL-17A and IFN-γ. The samples were acquired via FACS and analyzed with FlowJo Software V.10 (Tree Star).

### Immunohistochemistry

RNAscope in situ hybridization (Advanced Cell Diagnostics, ACD) was performed to detect IL-38 mRNA expression in PyMT tumors. Formalin-fixed paraffin-embedded tissue sections were pretreated before hybridization according to RNAscope Multiplex Fluorescent V.2 assay’s instructions. For the hybridized step, probes targeting murine RNA IL1F10 (ACD #524771) were incubated for 2 hours followed by three cycles of amplification. Gene expression was detected with RNAscope Multiplex Fluorescent Detection Reagent Kit V.2 (ACD) in combination with Opal dyes (Akoya Bioscience). Thereafter, tumor sections were stained with murine antibodies against Pan-Cytokeratin (Abcam) and Opal dyes (Akoya Bioscience) following the manufacturer’s instructions. Human Tissue Microarray specimens were purchased from TriStar (TriStar Technology Group, Washington, DC, USA) for Triple Negative Breast Cancer (catalog number: 69572270), Herceptin Eligible Breast Cancer (catalog number: 69571139) and Relapsed ER+ Breast Cancers (catalog number: 69572075-1621). PyMT tumors and human tissue microarrays (TMAs) were stained with Opal Fluorescence IHC Kits (Akoya Bioscience) in the BOND-RX Multiplex IHC Stainer (Leica Biosystems). Human TMAs were stained with primary antibodies against IL-38 (Thermo Fisher), CD3 (Ventana), CD8 (Dako), CD4 (Abcam), γδ-TCR (Santa Cruz), FOXP3 (Abcam), XCL1 (Atlas antibodies), and XCR1 (Cell Signaling). For PyMT tumors, the following murine primary antibodies were used: CD3 (Abcam), CD8 (Cell signaling), CD4 (Cell Signaling), Hes1 (Cell Signaling), MHC-II (Invitrogen), and F4/80 (Cell Signaling). In all sections, nuclei were counterstained with 4′,6-diamidino-2-phenylindole (DAPI). Vectra3 automated quantitative pathology imaging system was used to acquire the image slides at 20× magnification and images were analyzed with inForm V.2.6 software (Akoya Bioscience). TMAs cores were analyzed based on quality and tissue integrity after staining. Based on that, 167 individual cores were suitable for analysis.

### Whole transcriptome RNA sequencing

PyMT tumors were dissociated as described above and RNA was isolated using TRIzol Reagent (Life Technologies), followed by RNA purification with RNA Clean and Concentrator-5 (Zymo Research). RNA quality was evaluated in Agilent TapeStation 4150 with RNA Screen Tape (Agilent) and concentration was measured by Qubit HS RNA Assay Kit (Thermo Fisher Scientific). Thereafter, 500 ng of total RNA was taken for complementary DNA library preparation using QuantSeq 3’ mRNA-Seq Library Prep Kit FWD with 12 nt Unique Dual Indices (Lexogen) according to the manufacturer’s instructions. DNA quality and quantification were evaluated with High Sensitivity D1000 ScreenTape (Agilent) and Qubit dsDNA HS Assay Kit (Thermo Fisher Scientific), respectively. Libraries were sequenced (single-read, 75 cycles) using High Output Kit V.2 (Illumina) on a NextSeq 2000 sequencer (Illumina).

### Differential expression and GSEA analysis

RNA sequencing data was analyzed using the QuantSeq data analysis pipeline from BlueBee Genomics. Genes significantly regulated in the absence of IL-38 and on γδ-TCR blockage were analyzed using the Molecular Signatures Database using Gene Set Enrichment Analysis V.4.3.2 provided by the GenePattern Platform.

### Analysis of human publicly available data sets

Clinical data and gene expression from a publicly available data set of human breast cancer^22^ were downloaded from the cBioPortal for Cancer Genomics^23^ and analyzed using GraphPad Prism V.9.

### Statistical analysis

Data are presented as mean±SEM. Statistically significant differences between the two groups were calculated using paired or unpaired two-tailed Student’s t-test by Mann-Whitney. For multiple comparisons, multiple t-tests or one-way analysis of variance was used followed by appropriate post-correction analysis. D’Agostino and Pearson omnibus normality tests were performed to determine data distribution and variance. Statistical survival analysis was determined via log-rank test and correlation using the Spearman test. Statistical analysis was performed with GraphPad Prism V.9 and p values<0.05 were considered significant.

### Data availability

The transcriptomic data sets generated during and/or analyzed during the current study are available at GEO: GSE239398. The atomic coordinates of Fab e04 in complex with mIL-38 have been deposited in the Protein Data Bank (Accession No. 8Q3J). All other data sets generated during and/or analyzed during the current study are available from the corresponding author upon reasonable request.

## Results

### Elevated antitumor immunity in IL-38-deficient murine mammary carcinoma

**Figure 1:**
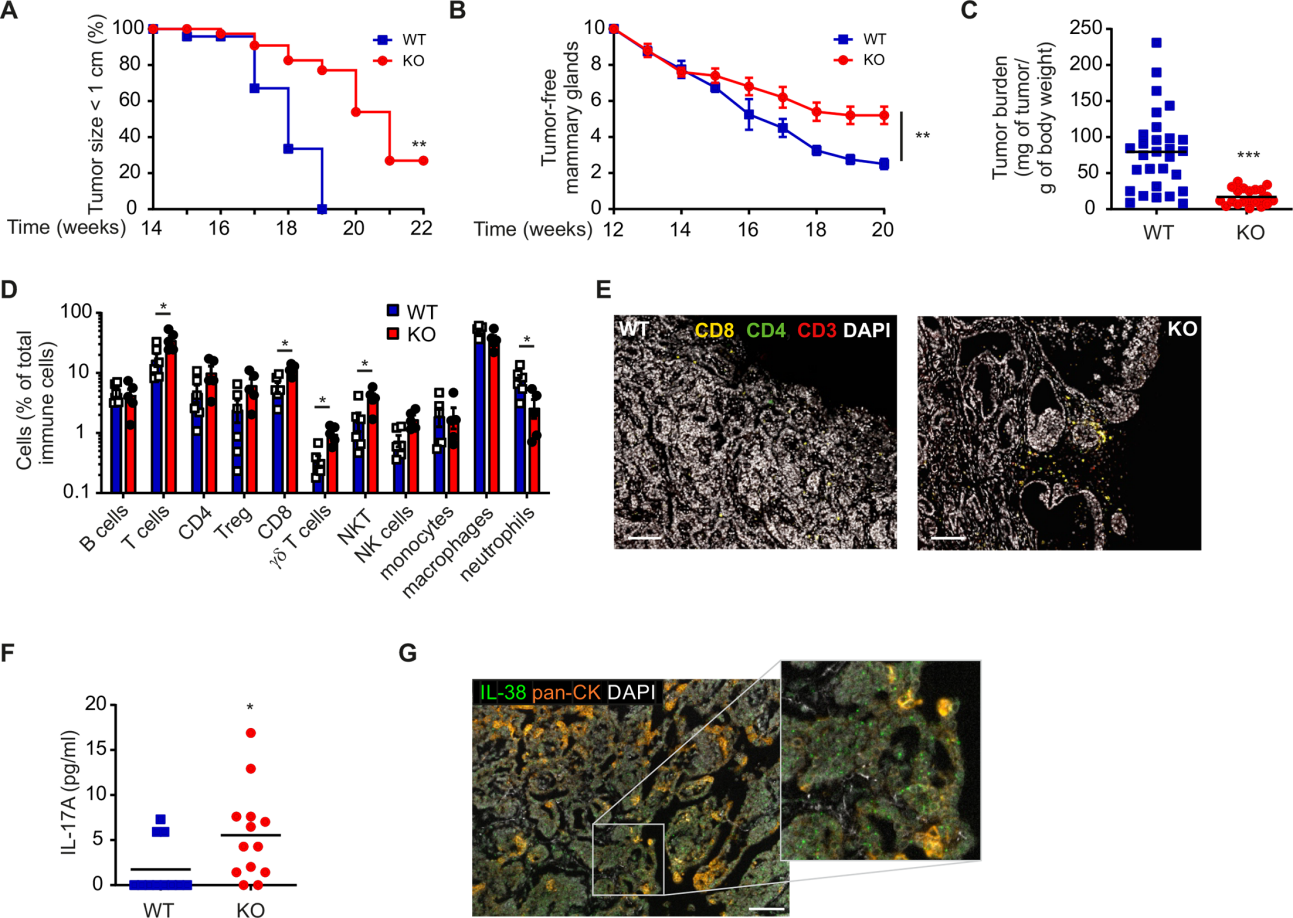
IL-38-deficient mice show delayed tumor development and increased T-cell infiltrates into tumors. (**A–G**) Polyoma middle T oncogene (PyMT) mice were wildtype (WT) or were crossed into an IL-38-deficient background (KO), and tumor development was monitored. (**A**) The survival analysis indicates the time point when the first mammary tumor reached a diameter of 1 cm; n=10 for each genotype. (**B**) The number of tumor-free mammary glands (of 10 in total) at week 20 is shown. (**C**) Tumor burden at week 20 is displayed. Data are means±SEM of 10 individual animals each. (**D**) Major immune cell populations infiltrating PyMT tumors were analyzed by flow cytometry at week 20. Data are means±SEM of six individual animals each. (**E**) PyMT mammary tumors were stained with antibodies against the markers indicated. Nuclei were counterstained with DAPI. Scale bars indicate 100 µm. (**F**) IL-17A expression levels in PyMT tumors were determined by cytometric bead array. (**G**) In situ hybridization by RNAscope showing the expression of IL-38 (green) in PyMT mammary tumors. Epithelial cells are marked with pan-cytokeratin antibody (orange). Nuclei were counterstained with DAPI. The scale bar indicates 100 µm. *p<0.05, **p<0.01, ***p<0.001; p values were calculated using unpaired multiple t-tests with FDR correction.DAPI, 4′,6-diamidino-2-phenylindole; FDR, false discovery rate; IL, interleukin; NK cells, natural killer cells; NKT cells, natural killer T cells; SDS-PAGE, sodium dodecyl-sulfate polyacrylamide gel electrophoresis.

To test if interfering with IL-38 affects tumor immunity, we employed an immunologically challenging model of cancer, the transgenic PyMT carcinoma model, with high relevance for human disease. The PyMT model is characterized by low-grade antitumor inflammation, such as a poor response to anti-programmed cell death protein-1 (PD-1) immune checkpoint blockade.^24 25^ Thus, only strong modulators of tumor immunity affect tumor growth in this model. When comparing tumor growth in WT and IL-38 KO PyMT mice, we observed a marked delay in the time until IL-38 KO tumors reached 1 cm in diameter (figure 1A). At week 20, the number of tumor-burdened mammary glands was lower in IL-38 KO PyMT tumors (figure 1B), as was the overall tumor burden (figure 1C). Flow cytometric immune profiling revealed an increase in lymphocytes, including CD8+ T cells, γδ T cells, and natural killer T cells (NKT cells), but not regulatory T cells, in IL-38 KO tumors (figure 1D,E), suggesting enhanced immune control. Confirming the prominent role of IL-38 in restricting IL-17 production, we noticed increased IL-17 protein levels in IL-38 KO tumors, even though we considered the low IL-17 levels that were detected to be likely biologically irrelevant (figure 1F). IL-38 was mainly expressed in cancer cells, which was determined at mRNA level due to the absence of specific IL-38 antibodies for mouse tissues (figure 1G). Overall, these data indicate that IL-38 restricts antitumor immunity in mammary carcinoma.

### IL-38 blockade limits mammary tumor growth

**Figure 2:**
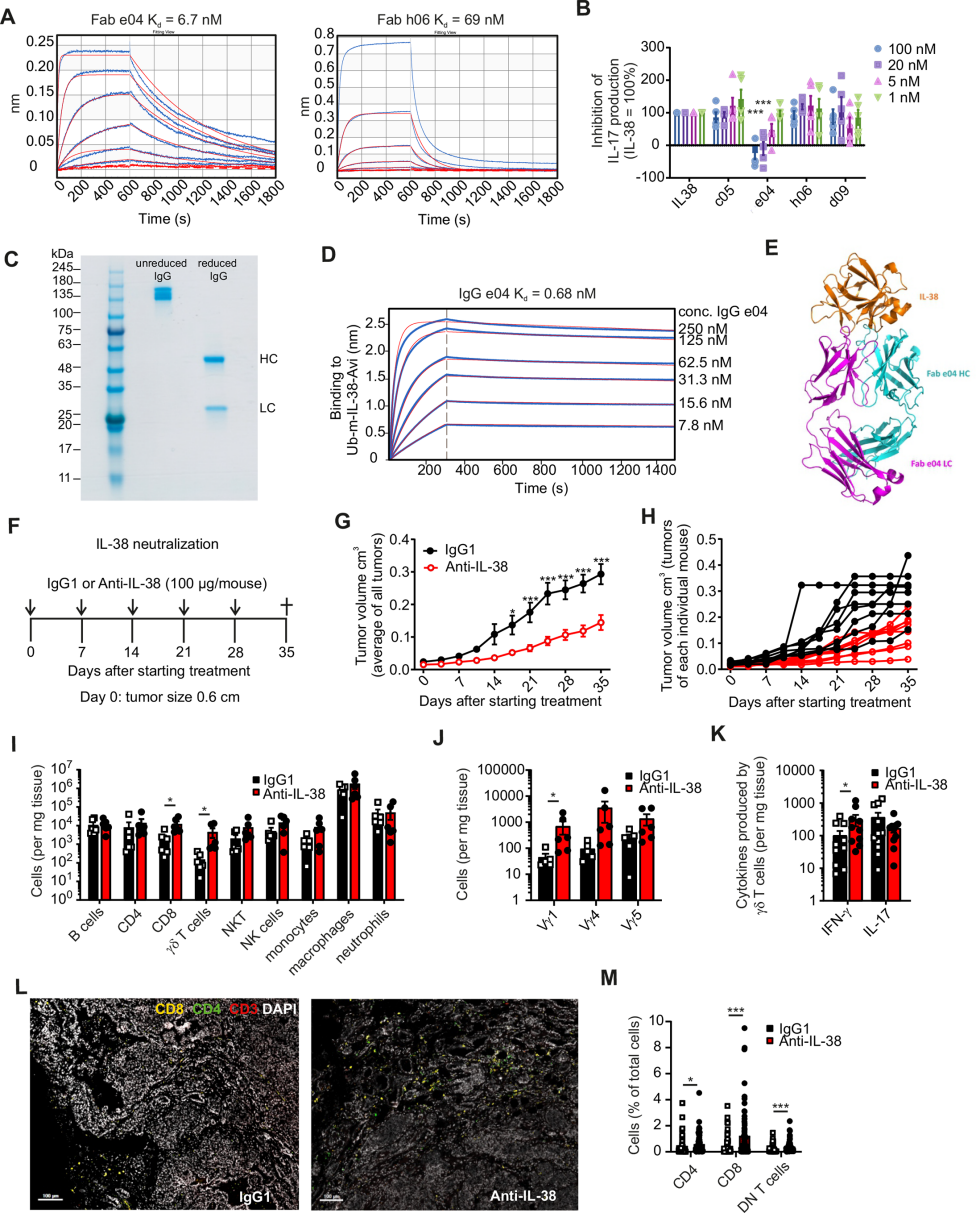
IL-38 neutralization slows down tumor development and increases T-cell infiltrates into tumors. (**A**) Kinetic analysis and dissociation constants (Kd) of Fabs e04 and h06 selected by phage display binding to mouse IL-38 determined by biolayer interferometry. (**B**) Mouse splenic γδ T cells were seeded on anti-CD3 coated plates and treated with IL-1 and IL-23 + IL-38 and Fab fragments putatively recognizing cells murine IL-38. IL-17 levels in supernatants were analyzed after 5 days. Unrelated Fab fragments c05 and d09 were used as negative controls. Data are from four independent experiments. (**C**) SDS-PAGE analysis of purified e04 IgG. (**D**) Binding profile of e04 IgG and mouse IL-38 determined by biolayer interferometry. (**E**) Crystal structure of Fab E04 binding to recombinant IL-38. (**F–K**) Polyoma middle T oncogene (PyMT) wildtype mice (n=8) were treated with either IgG1 isotype control or anti-IL-38 antibodies (100 µg/mouse) once a week. (**F**) Tumor growth was monitored when the first tumor reached 0.6 cm in diameter. (**G**) The average tumor burden is displayed per group and (**H**) for each individual mouse. (**I**) The overall immune cell profile (n=6), (**J**) γδ T-cell subsets (n=5–6) and (**K**) intracellularly produced cytokines (n=10–11) were determined by flow cytometry after 5 weeks of treatment. (**L**) Representative images of PyMT tumors stained for CD8, CD4 and CD3 and (**M**) quantification of indicated cells is shown. CD3+CD4− CD8− double negative (DN) contain γδ T cells. Nuclei were costained with DAPI. Scale bars indicate 100 µm. Data are shown as means±SEM. *p<0.05, **p<0.01, ***p<0.001; p values were calculated using unpaired multiple t-tests with FDR correction. DAPI, 4′,6-diamidino-2-phenylindole; FDR, false discovery rate; IL, interleukin; IFN, interferon; KO, knockout; NK cells, natural killer cells; NKT cells, natural killer T cells; Treg, regulatory T cells.

To investigate if acute blockade of IL-38 mimics the impact of genetic IL-38 ablation on tumor growth, we generated an IL-38 neutralizing antibody using phage display. One Fab fragment (e04) was identified to bind to murine IL-38 with high affinity (6.7 nM) (figure 2A), and neutralized the inhibitory effect of IL-38 on IL-17 production by γδ T cells (figure 2B), which we had identified previously.^10^ Reformatting the Fab fragment to a full-length IgG1 antibody improved the Kd to 0.68 nM (figure 2C,D). Crystallization of the Fab fragment in complex with IL-38 was achieved to gain insights about critical interaction sites (figure 2E, online supplemental figure S1). When using this antibody in a therapeutic setting in PyMT WT mice once the first tumor had reached a size of 0.6 cm in diameter (figure 2F), we noticed a marked delay in tumor development (figure 2G,H). This was accompanied by an increased abundance of CD8+ T cells and γδ T cells in the tumors (figure 2I), while other microenvironmental cell populations were not significantly affected (online supplemental figure S2A). Among γδ T cells, an increase of all tested subsets was noticed, which reached significance only for the Vγ1 subset (figure 2J), which produces mainly IFN-γ.^26^ Accordingly, IFN-γ rather than IL-17-producing γδ T cells were increased on IL-38 neutralization (figure 2K). Histological confirmation of an increase in γδ T cells was limited due to the unavailability of a specific antibody for the murine γδ TCR. However, we noticed an increase in double negative T cells in the mammary tumors, of which γδ T cells are a major subset (figure 2L,M; online supplemental figure S2B). These data suggest that interfering with IL-38 might be beneficial to limit tumor growth.

### IL-38 blockade synergizes with chemotherapy

**Figure 3:**
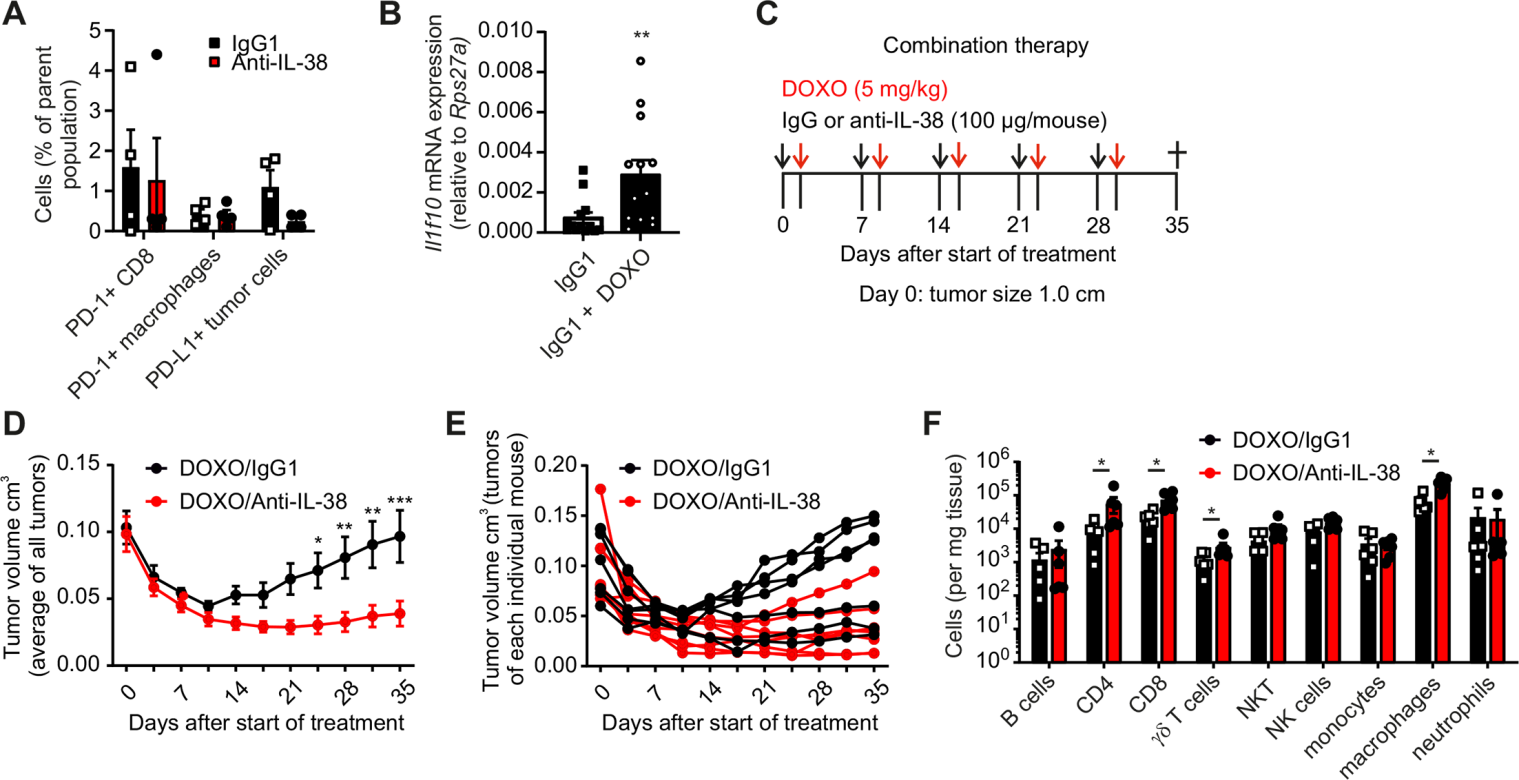
IL-38 blockade prevents tumor progression after chemotherapy. (**A**) In tumors from PyMT mice treated with either IgG1 or anti-IL-38 antibodies, relative numbers of CD8+ T cells, macrophages, and tumor cells which express PD-1 or PD-L1 were analyzed by flow cytometry (n=4). (**B**) *II1f10* expression was determined by quantitative PCR in tumors from PyMT wildtype mice treated with IgG1 in combination with doxorubicin (DOXO) (n=13). (**C–F**) PyMT mice were treated with either IgG1 or anti-IL-38 (100 µg/mouse) followed by combinatory treatment with doxorubicin (5 mg/kg) the day after. (**C**) Tumor growth was monitored once the first tumor reached 1.0 cm size in diameter (n=8). (**D**) The average of tumor burden is displayed per group and (**E**) for each individual mouse. (**F**) The immune cell profile was determined by flow cytometry at the endpoint (n=7). Data are shown as means±SEM. *p<0.05, **p<0.01, ***p<0.001; p values were calculated using unpaired multiple t-tests with FDR correction, with the exception of (**B**) (Student’s t-test). FDR, false discovery rate; IL, interleukin; mRNA, messenger RNA; NK cells, natural killer cells; NKT cells, natural killer T cells; PD-1, programmed cell death protein-1; PD-L1, programmed death-ligand 1; PyMT, polyoma virus middle T oncoprotein.

Given the immunostimulatory potential of neutralizing IL-38, we asked if IL-38 blockade might sensitize to other forms of therapy. We did not observe an impact of IL-38 neutralization on the expression of the PD-1/PD-L1 immune checkpoint (figure 3A). Therefore, we refrained from testing IL-38 in combination with immune checkpoint blockade. IL-38 is secreted by dying cells, which can also be monitored at the transcriptional level.^12^ Indeed, treating PyMT mice with the chemotherapeutic drug doxorubicin increased IL-38 expression in the tumor (figure 3B). When combining IL-38 blockade with doxorubicin treatment in a late-stage cancer therapeutic setting starting at a tumor size of 1 cm (figure 3C), we observed that combination of IL-38 blockade with doxorubicin limited tumor progression, whereas chemotherapy alone did not (figure 3D,E). This was, again, accompanied by an increased abundance of T cells, including CD8+ T cells and γδ T cells (figure 3F), similar to IL-38 blockade alone or IL-38 ablation (figures1D  2I).

### IL-38-dependent reduction in tumor growth is reversed by targeting γδ T cells

**Figure 4:**
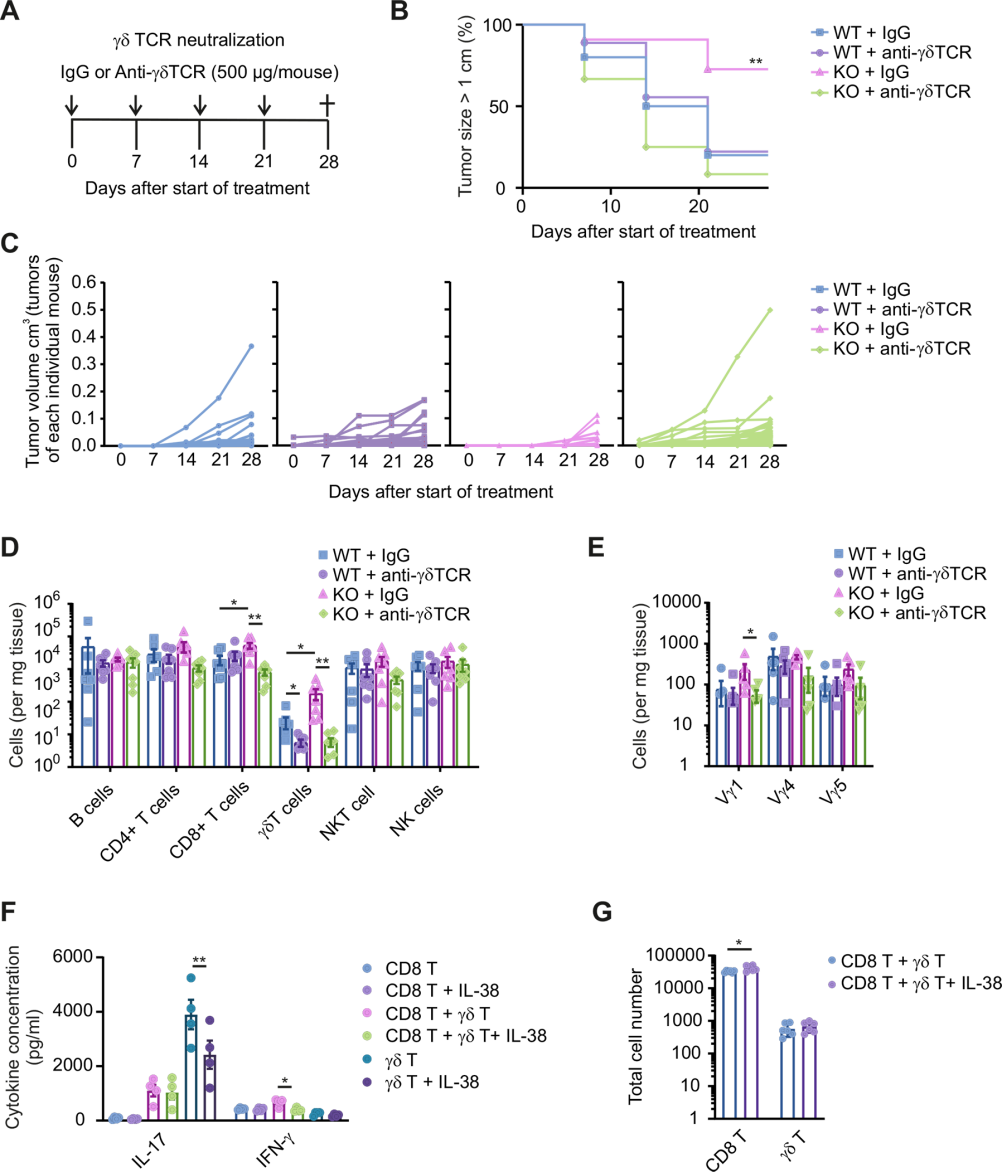
Targeting the γδ TCR abrogates the impact of IL-38 ablation. (**A–E**) PyMT IL-38 KO and WT were treated with either IgG or γδ-TCR blocking antibodies (**A**) Tumor growth was monitored once mice were 13 weeks old. (**B**) The survival analysis indicates the time point when the first mammary tumor reached a size of 1 cm in diameter (n=10) for each group. (**C**) The average tumor burden of the individual mice group is displayed. (**D**) Total immune cell profiles (n=7–9) and (**E**) γδ T-cell subsets (n=6–7) were determined by flow cytometry at the endpoint. (**F,G**) γδ T cells and CD8+ T cells were FACS-sorted from the spleen and cultured for up to 5 days. (**F**) Levels of IL-17 and IFN-γ were measured by cytometric bead array (n=4) as well as (**G**) T-cell numbers by flow cytometry (n=6) at day 5 and represent two independent experiments. Data are shown as means±SEM. *p<0.05, **p<0.01, ***p<0.001; p values were calculated using unpaired multiple t-tests with FDR correction, with the exception of (**F**) (one-way analysis of variance). FACS, fluorescence-activated cell sorting; FDR, false discovery rate; IFN, interferon; IL, interleukin; KO, knockout; NK cells, natural killer cells; NKT cells, natural killer T cells; TCR, T-cell receptor; WT, wildtype.

IL-38 neutralization had a major impact on CD8+ T cells and γδ T cells. Since we previously observed that γδ T cells were primary targets of IL-38 during skin inflammation,^10^ we first asked for the role of these cells in our model. We used a γδ TCR neutralizing antibody that, rather than depleting γδ T cells, blocks signaling via the γδ TCR.^10 27^ We tested the effect of this antibody compared with the isotype control in WT versus IL-38 KO PyMT mice, from week 13 onwards for 5 weeks (figure 4A), when differences concerning tumor growth between WT versus KO tumors were not yet apparent (figure 1A). In this setting, blocking the γδ TCR reversed the reduced tumor growth in IL-38 KO mice, but did not affect WT mice (figure 4B,C). As expected, blocking the γδ TCR decreased γδ T-cell numbers in WT and IL-38 KO tumors (figure 4D), presumably through reducing activation-induced proliferation rather than depleting the cells, as their levels were largely unchanged in spleen and blood (online supplemental figure S2E,F). This decrease was most prominently observed for the Vγ1 subset, which was, again, increased on IL-38 ablation (figure 4E). Importantly, CD8+ T cell numbers were only reduced in IL-38 KO tumors on γδ TCR blockade (figure 4D). These data suggested a direct connection between γδ T cells and CD8+ T cells when interfering with IL-38 in tumors. However, co-culture experiments did not support this notion. While IL-38 effectively suppressed IL-17 production by splenic γδ T cells in vitro, and also limited the secretion of IFN-γ that was induced by co-culturing γδ T cells and CD8+ T cells (figure 4F), the number of CD8+ T cells was rather enhanced in such co-cultures (figure 4G). Thus, γδ T cells appeared to indirectly affect CD8+ T cell abundance in PyMT tumors.

### Inhibiting IL-38 increases cDC1 infiltration into PyMT tumors

**Figure 5:**
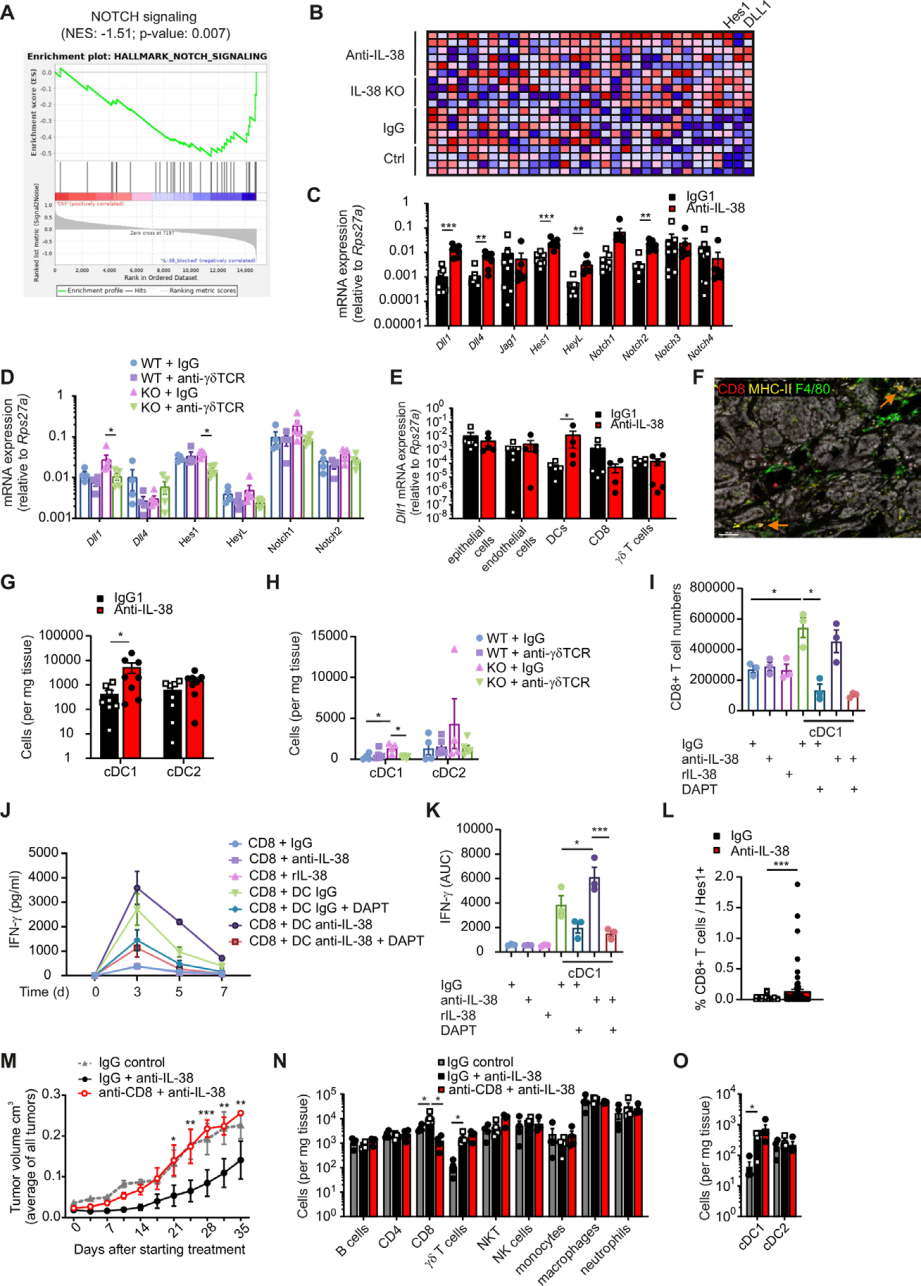
γδ T cells recruit cDC1 into tumors to supply Notch signals. (**A–D**) Total RNA was isolated from PyMT tumors after in vivo IL-38 neutralization and transcriptomes were analyzed by whole transcriptome sequencing analysis. (**A**) GSEA plot indicates an increase in Notch signaling on IL-38 neutralization. (**B**) The heatmap indicates the expression profile of Notch pathway genes in IgG and anti-IL-38 treated groups. (**C,D**) The expression of the indicated Notch pathway genes was analyzed by qPCR in PyMT tumors on IL-38 neutralization (n=7–8) and/or γδ-TCR blockage (n=4–6). (**E**) Epithelial cells, endothelial cells, DCs, CD8+ T cells and γδ T cells were isolated by FACS-sorting from PyMT tumors on in vivo IL-38 neutralization followed by *Dll1* expression determination by qPCR (n=5–6). (**F**) The representative image of a PyMT tumor stained for MHC-II (yellow), F4/80 (green) and CD8 (red) by PhenOptics is displayed. Scale bar=40 µm. Orange arrows mark MHC-II+CD8+ F4/80 low cDC1. (**G,H**) cDC1 and cDC2 cell numbers in PyMT tumors were analyzed by flow cytometry (**G,**) n=8; **H**), n=4–6). (**I–K**) Splenic CD8+ T cells were co-cultured with cDC1 isolated from IL-38 neutralized PyMT tumors for up to 7 days. During that time, cells were supplemented with IgG, anti-IL38 antibodies, or recombinant IL-38, with or without γ-secretase inhibitor DAPT. The data are representative of two independent experiments with n=3 each. (**I**) CD8+ T cell numbers were analyzed by flow cytometry at day 7. (**J, K**) IFN-γ levels were measured by cytometric bead array on days 3, 5 and 7, and AUC data for quantification (**J**) and individual concentrations (**K**) are shown. (**L**) Quantification of Hes1-expressing CD8+ T cells was determined from PyMT tumors stained for CD3, CD8, CD4 and Hes1 by PhenOptics. (**M–O**) PyMT WT mice were treated with isotype controls or anti-IL-38 antibodies in combination with anti-CD8 antibodies once the first tumor reached 0,6 cm in diameter (n=4). The average tumor burden (**M**) was analyzed for each group. (**N,O**) The immune cell profile was analyzed by flow cytometry at the endpoint. Data are shown as means±SEM. *p<0.05, **p<0.01, ***p<0.001. P values were calculated using unpaired multiple t-tests with FDR correction, with the exception of (**I, J**) (one-way analysis of variance) and (**L**) Student’s t-test. AUC, area under the curve; cDC, conventional dendritic cell; DC, dendritic cell; FACS, fluorescence-activated cell sorting; FDR, false discovery rate; GSEA, gene set enrichment analysis; IFN, interferon; IL, interleukin; KO, knockout; MHC, major histocompatibility complex; mRNA, messenger RNA; NES, normalized enrichment score; NK cells, natural killer cells; NKT cells, natural killer T cells; PyMT, polyoma virus middle T oncoprotein; TCR, T-cell receptor; WT, wildtype.

**Figure 6:**
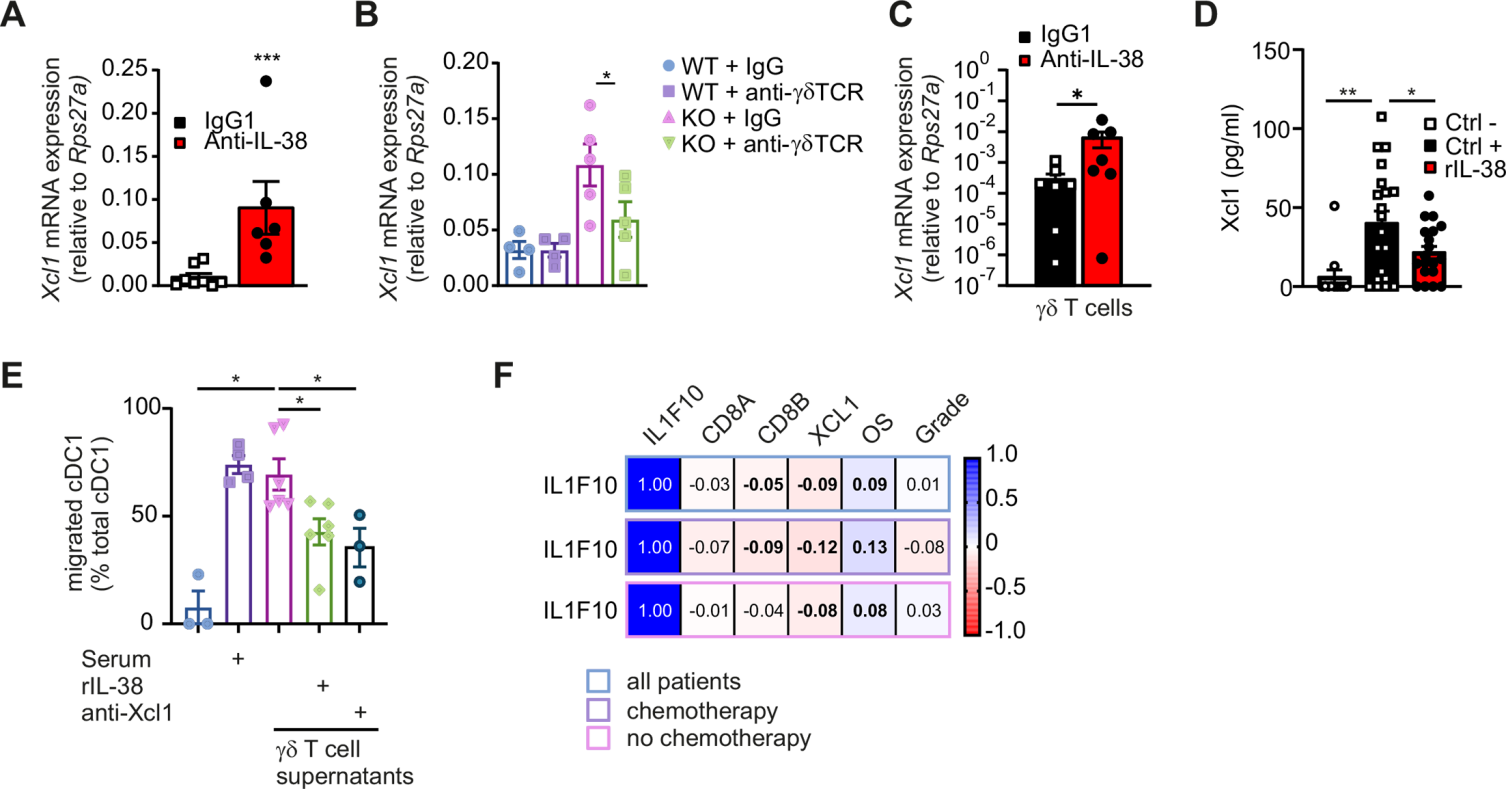
γδ T cells recruit cDC1 via Xcl1, dependent on IL-38. (**A–C**) PyMT IL-38 KO and WT were treated with either anti-IL-38 or anti-γδ-TCR antibodies with their respective IgG isotype controls. *Xcl1* expression was analyzed in the total RNA of PyMT tumors on (**A**) IL-38 neutralization (n=8) and (**B**) γδ-TCR blockage (n=4–5). (**C**) *Xcl1* mRNA expression was determined in FACS-sorted γδ T cells after in vivo IL-38 neutralization (n=8). (**D**) γδ T cells were isolated from the spleen and cultured for 24 hours in the presence or absence of recombinant IL-38 (rIL-38). The negative control (Ctrl−) corresponds to non-activated γδ T cells. Xcl1 levels were measured by ELISA. Data are from four individual experiments (n=20). (**E**) Dendritic cell enriched splenocytes added in transwell inserts of Boyden chamber were allowed to migrate towards pooled γδ T-cell supernatants from (**D**). Migrated cDC1 were determined by flow cytometry (n=3–6). Heat-inactivated FCS was used as a positive control and in one group the supernatant was pre-incubated with anti-Xcl1 antibodies. Data are representative of three individual experiments (n=3–6 each) (**F**) The METABRIC data set was used to calculate the correlation (Spearman r values are shown) between *IL1F10*, CD8+ T cells and *XCL1* expression in patients with mammary carcinoma. Statistically significant differences are indicated by bold numbers. Data are shown as means±SEM. *p<0.05, **p<0.01, ***p<0.001; (**B,E**) one-way analysis of variance and (**A,C,D**) Student’s t-test were used. cDC, conventional dendritic cell; FACS, fluorescence-activated cell sorting; FCS, fetal calf serum; IL, interleukin; KO, knockout; mRNA, messenger RNA; PyMT, polyoma virus middle T oncoprotein; TCR, T-cell receptor; WT, wildtype.

Expansion of γδ T cells and CD8+ T cells on interfering with IL-38 was only seen locally in tumors, not in spleen or blood (online supplemental figure S2G-J), indicating that local rather than systemic expansion of CD8+ T cells was required. To identify potential mechanisms, whole transcriptomes of PyMT tumors with or without IL-38 neutralization were combined with transcriptomes of WT versus IL-38 KO tumors. While there was high heterogeneity between samples, as expected due to the diverse etiology of individual PyMT tumors in the model, signaling via the Notch pathway emerged as a common principle on inhibiting IL-38 either genetically or via a neutralizing antibody compared with the WT or isotype control (figure 5A,B; online supplemental table S1). This was validated at the individual gene level by qPCR (figure 5C). When comparing the expression of these genes in tumors from WT and IL-38 KO mice with or without γδ TCR blockade, a consistent pattern emerged for the Notch ligand Delta like canonical Notch ligand 1 (*Dll1*) and the downstream mediator Hairy and Enhancer of split-1 (*Hes1*), which were both induced in IL-38 KO tumors, but not once the γδ TCR was blocked (figure 5D, online supplemental figure S3A). Interestingly, cDCs are known to activate CD8+ T cells by Notch signaling.^28^ Indeed, *Dll1* was upregulated on cDCs sorted from IL-38 neutralized versus control tumors compared with other cells in the tumor microenvironment (figure 5E). In contrast, the receptors Notch 1 and 2 were not altered in FACS-sorted TCD8 cells (online supplemental figure S3B,C). Moreover, cDC1, the subset particularly important for CD8+ T cell activation, were only observed in IL-38 neutralized tumors, but not in control tumors, by immunofluorescence analysis (figure 5F), and an increased infiltration of cDC1 in IL-38 neutralized and KO tumors was observed at a quantitative level, but not when the γδ TCR was blocked (figure 5G,H). Thus, γδ T cells might recruit cDC1 into PyMT tumors to promote CD8+ T cell activation. We next tested if the potential of cDC1 to activate CD8+ T cells was altered once IL-38 was blocked. We sorted cDC1 from IL-38 neutralized PyMT tumors, since the low numbers that could be isolated from control tumors was not sufficient, and co-cultured them with splenic WT CD8+ T cells. To analyze the role of the Notch pathway, the γ-secretase inhibitor DAPT was used.^29^ cDC1 increased both, proliferation and IFN-γ production by CD8+ T cells as compared with controls, which was strongly inhibited in the presence of DAPT (figure 5I–K). This was independent of IL-38 neutralization and/or the addition of recombinant IL-38, even though there was sustained IFN-γ production at higher levels when IL-38 was neutralized (figure 5J,K). These data suggest that increased recruitment of cDC1 promoted CD8+ T cell activation on IL-38 neutralization. The role of Notch signaling in this process was further supported by increased expression of Hes1 in CD8+ T cells in tumors on IL-38 blockade (figure 5L). To substantiate the role of CD8+ T cells on IL-38 neutralization, we depleted CD8+ T cells in PyMT mice treated with anti-IL-38 antibodies. The CD8+ T cell-depleted group showed a strong increase in tumor burden when compared with mice in which IL-38 was blocked alone, reaching levels similar to mice where IL-38 was not blocked (figure 6M). Immune cell profile analyses revealed that CD8+ T cells were efficiently depleted, but γδ T cell and cDC1 numbers were not affected (figure 6N,O). These data support the view that CD8+ T cells operate downstream of γδ T cell and cDC1 on IL-38 blockade.

### γδ T cells recruit cDC1 via Xcl1

cDC1 express the chemokine receptor Xcr1, with Xcl1 being its major ligand.^30^ We wondered if γδ T cells might produce Xcl1 in tumors to recruit cDC1. *Xcl1* expression was increased in PyMT tumors when IL-38 was blocked or ablated, while neutralizing the γδ TCR reduced *Xcl1* expression in IL-38 KO tumors (figure 6A,B). FACS-sorted γδ T cells from tumors showed increased *Xcl1* expression on IL-38 neutralization rather than other cells (figure 6C; online supplemental figure S2K). Moreover, isolated γδ T cells from WT spleens produced increased levels of Xcl1 on γδ TCR-dependent activation, which was suppressed by recombinant IL-38 (figure 6D). Supernatants of activated γδ T cells induced cDC1 migration in Boyden chamber assays, which was reduced when γδ T cells were pretreated with IL-38 or when Xcl1 was neutralized with an antibody (figure 6E). These data show that IL-38 regulates Xcl1 secretion from γδ T cells, and are consistent with the view that IL-38 blockade propagates cDC1 recruitment into tumors by γδ T cell-derived Xcl1, while cDC1 in turn activate CD8+ T cells via Notch signaling to improve tumor immune control (online supplemental figure S4). Interestingly, IL-38 (*IL1F10*) expression correlated negatively with the expression of *XCL1* and *CD8* in the METABRIC data set,^22^ supporting the proposed mechanism (figure 6F). This negative correlation was more apparent in patients who had received chemotherapy, indicating a sensitizing effect towards the IL-38 system as suggested by our murine data (figure 3).

### IL-38 is associated with T-cell infiltration and survival in human mammary carcinoma

**Figure 7:**
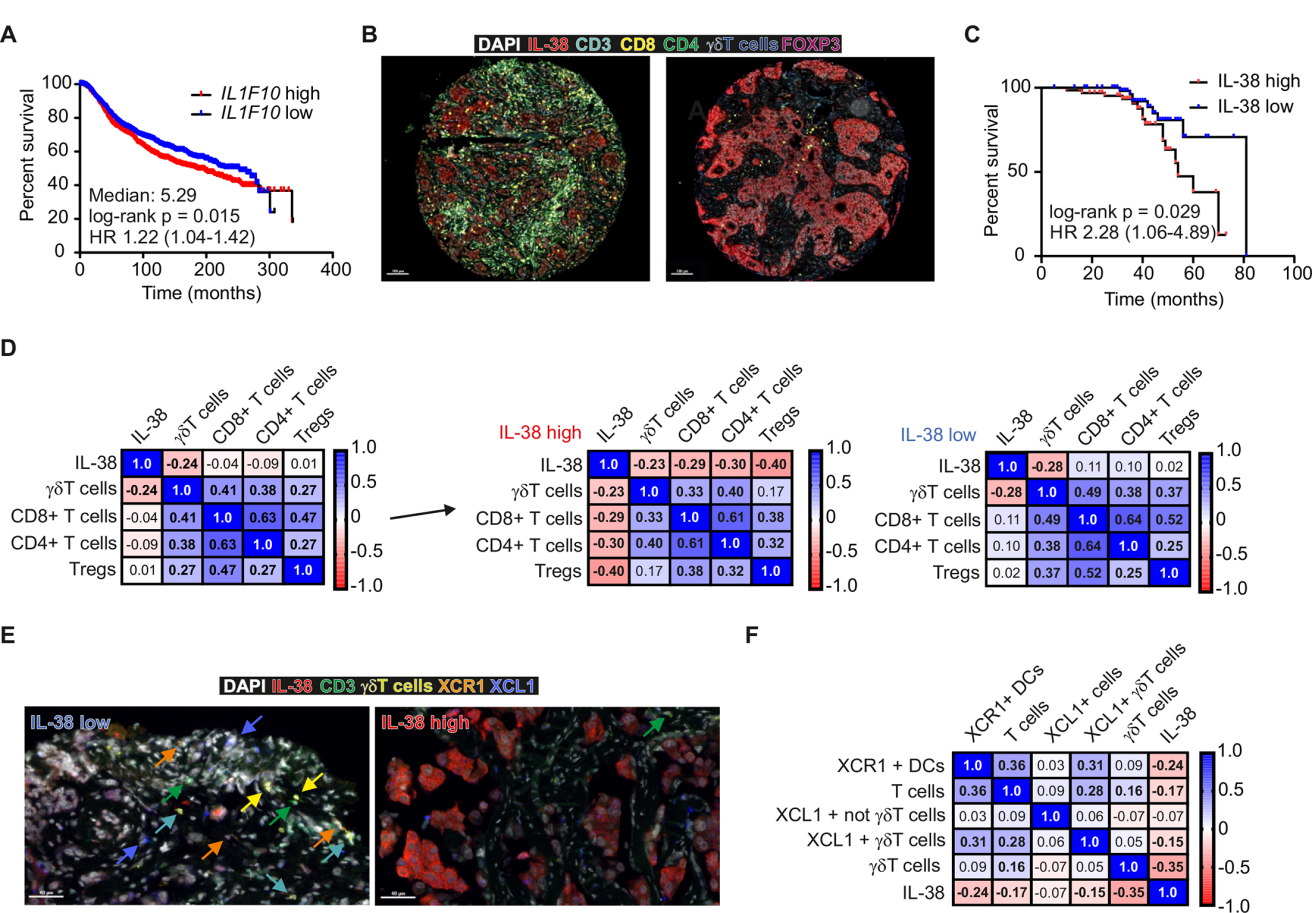
IL-38 correlates with survival and T cells in mammary cancer. (**A–D**) The METABRIC data set,^22^ (Curtis *et al*, 2012 Nature) was used to analyze the impact of IL-38 (*IL1F10*) on patient with mammary carcinoma survival. (**B–F**) Human mammary carcinoma tissue microarrays (167 cores) were analyzed for expression of IL-38, T-cell markers, XCR1 and XCL1. (**B**) Representative images of IL-38 low and high tissue cores, (**C**) patient survival and (**D**) correlation (Spearman r values are shown) of IL-38 in high and low IL-38 expressing tumors with T-cell subsets are shown. Statistically significant differences are indicated by bold numbers. (**E**) Representative images of XCR1+DCs and XCL1-expressing cells and (**F**) the correlation (Spearman r values are shown) with IL-38 expression were analyzed. Statistically significant differences are indicated by bold numbers. DAPI, 4′,6-diamidino-2-phenylindole; DCs, dendritic cells; IL, interleukin; Treg, regulatory T cells.

We further explored a potential role for IL-38 in human breast cancer. In the METABRIC data set,^22^ expression of *IL1F10,* encoding IL-38 negatively correlated with survival in patients with breast cancer (figure 7A). There was a tendency for the stronger negative association of IL-38 with survival in triple-negative breast cancer (claudin-low, normal-like and basal-like subtypes) (online supplemental figure S5B). These findings were further investigated by analyzing IL-38 protein expression and comparing it to T-cell subset abundance in breast cancer tissue microarrays. As in mice, IL-38 was mainly expressed in cancer cells (figure 7B, online supplemental figure S5A), and high IL-38 expression negatively correlated with survival (figure 7C). Importantly, high IL-38 expression also negatively correlated with overall T-cell infiltrates. Moreover, a positive correlation between CD8+ T cells and γδ T cells was observed. However, in IL-38-low tumor cores, only the positive correlation between CD8+ T cells and γδ T cells, and the negative correlation between IL-38 and γδ T cells remained, which may indicate a particular sensitivity of γδ T cells for IL-38 (figure 7D). Further analysis in the breast cancer tissue microarrays revealed a positive correlation of cDC1 (XCR1+DCs) with XCL1-producing γδ T cells. Moreover, both cDC1 and XCL1-producing γδ T cells negatively correlated with IL-38, but positively correlated with T cells (figure 7E,F). Overall, these data support the notion that IL-38 limits the abundance of antitumor immune cells also in humans.

## Discussion

Our data suggest that blocking IL-38 signaling may be of interest to trigger antitumor immune responses. Different approaches have been developed to overcome immune suppression and to re-activate an effective antitumor immune response. The success of such therapies would be reflected by an altered quantity and quality of the tumor immune infiltrate, whereby enhanced infiltrates of cytotoxic lymphocytes such as CD8+ T cells, NK cells and γδ T cells are associated with a favorable prognosis.^31 32^ Of the immunotherapeutic options explored in clinical studies, immune checkpoint inhibitors have shown remarkable efficacy.^33^ However, a significant group of patients does not react to current immunotherapy, including immune checkpoint inhibitors. Efforts to improve this situation focus, among others, on identifying new immunoregulatory pathways in the tumor microenvironment to be used for combinatory therapy and/or as a factor to predict whether patients respond to immune checkpoint blockade. Previous findings that IL-38 released from dying tumor cells blocks inflammatory macrophage activation suggested a potential role of IL-38 in limiting antitumor immunity.^12^ Indeed, IL-38 overexpression reduced CD8+ T cells infiltration in subcutaneously (s.c.) implanted lung tumors, and IL-38 blockade increased immune cell infiltration, leading to better tumor growth control in s.c. implanted EMT6 murine breast cancer and B16.F10 murine melanoma models.^18 34^ The present study shows that both, genetic ablation and pharmacological blockade of IL-38 in models where tumors develop endogenously over a longer period of time, is equally effective in promoting potentially cytotoxic immune cell infiltration. Of note, the PyMT tumor model is largely resistant to PD-1/PD-L1 immune checkpoint blockade. Therefore, IL-38 blockade may be of benefit for patients not responding to current strategies that interfere with immune checkpoints.

Among the cellular markers suggesting an increased antitumor immune milieu, CD8+ T cell, cDC1 and γδ T cells were dominant. γδ T cells have been classified as the most favorable prognostic cell population across a multitude of human tumors.^31^ γδ T cells possess unconventional T-cell features that include major histocompatibility complex-dependent antigen presentation and using NK cell receptors to directly kill target cells.^35 36^ The antitumor function of γδ T cells is mainly associated with their cytotoxic potential and cytokine production, but also tumor-promoting functions of γδ T cells have been described.^37 38^ Subtypes of γδ T cells can be classified based on TCR-γ-chain variable region (Vγ) expression. Among the Vγ subgroups tested in the present study, Vγ1-expressing γδ T cells were most consistently enriched on neutralization of IL-38. These γδ T cells produce large amounts of IFN-γ,^26^ which might increase immunosurveillance of γδ T cells in vivo. Indeed, IFN-γ producing γδ T cells display potent cytotoxic effects, leading to delayed tumor growth in the murine B16 melanoma and chemical carcinogen methylcholanthrene models.^39 40^ In humans, recent studies have connected Vδ1-expressing γδ T cells with protective immunity.^41 42^ Intriguingly, antitumor functions in murine γδ T cells were most closely mirrored by Vγ1+ and Vγ7+ cells.^42^ These findings combined with our neutralization experiment suggest that IL-38 blockade might improve antitumor responses, particularly via Vγ1+ γδ T cells.

The receptor through which IL-38 predominantly signals to limit protective immunity remains elusive. Three receptors have been proposed for IL-38, including IL-1 receptor (IL-1R1), IL-36 receptor (IL-1R6), and IL-1 receptor accessory protein-like 1 (IL-1RAPL1; IL-1R9). γδ T cells isolated from PyMT tumors expressed IL-36 receptor (online supplemental figure S3D). Even though IL-1RAPL1 gene expression was not detected in γδ T cells isolated from PyMT tumors, probably due to low expression combined with low input material, our previous studies suggested IL-1RAPL1 as a major IL-38 receptor in γδ T cells. Interestingly, *IL1RAPL1* expression correlates with improved prognosis in patients with mammary carcinoma (online supplemental figure S5C) and with the γδ T-cell marker *ZBTB16* (online supplemental figure S5D). *ZBTB16* expression, in turn, correlated with survival in the METABRIC data set (online supplemental figure S5E), indicating that the presence of cells expressing this transcription factor, including γδ T cells, promotes patient survival. Subgrouping patients based on the combined expression of *ZBTB16* and *IL1RAPL1*, moreover, indicates that patients expressing high *IL1RAPL1* and *ZBTB16* levels have a superior survival probability compared with patients expressing low levels of both markers (online supplemental figure S5E). These data may suggest that a high expression of *IL1RAPL1* in γδ T cells is beneficial for patient survival, and thus point towards IL-38 acting on this receptor in mammary tumors.

IL-38 acting on γδ T cells suppressed the production of Xcl1. Xcl1 is produced by lymphocytes, including γδ T cells, CD8+ T cells, CD4+ T cells, NK, and natural killer T cells (NKT cells), and its receptor Xcr1 is selectively expressed by cDC1.^43^ Xcl1 secreted by γδ T cells in the intestinal lamina propria promotes Xcr1+ cDC1 migration to the mesenteric lymph node inducing anti-CD3 oral tolerance.^44^ cDC1 specialize in antigen cross-presentation to naive CD8+ T cells.^45^ These CD8+ T cells most likely then sense tumor neoantigens or tumor-associated antigens, which have been identified in the PyMT model. Overall, tumorigenesis triggered by the PyMT oncogene is coupled to additional genetic modifications, such as gene amplification and mutations. These include mutations in the receptor tyrosine phosphatase *Ptprh* that triggers the constitutive activation of the epidermal growth factor receptor and *Mtor*.^46^ The precise nature of tumor antigens recognized by CD8+ T cells in our study requires additional deeper analyses.

We observed an increase of cDC1, mirroring an enhanced CD8+ T cell abundance, which might be functionally linked via Notch signaling on IL-38 ablation. The canonic Notch pathway is regulated by the Notch receptors (Notch1-4) and their ligands Jagged1, Jagged2, Dll1, Dll3 and Dll4. Consequentially, a sequence of proteolytic events releases the Notch intracellular domain that in turn translocates to the nucleus and in association with the recombination signal binding protein for immunoglobulin kappa J region complex triggers the expression of, among others, *Hes1* and *Hairy* and *Enhancer-of-split* related with YRPW motif (*Hey*).^47 48^ Previous studies report that in acute influenza virus infection, the Notch pathway promotes the generation of CD8+ effector T cells. Moreover, Notch ligands Dll1 and Jag1 were upregulated on migratory DCs from the lung and draining lymph nodes on influenza infection, which in turn primed naïve CD8+ T cells to assemble a specific virus response.^49^ In intracellular Notch1-expressing mice, an increase in cytotoxicity of CD8+ T cells mediated by IFN-γ and granzyme B production was observed, which delayed tumor growth in the syngeneic murine Lewis lung carcinoma model.^50^ Genetic depletion of Dll1 in CD11c+ murine cells suppressed effector CD8+ T cell activation leading to tumor progression in lung and pancreatic tumors.^51^ These data support our findings of a Notch-dependent activation of CD8+ T cells by cDC1 from PyMT tumors. Both cDC1 and CD8+ T cells were increased dependent on IL-38 and γδ T cells. We therefore propose a model where activated γδ T cells recruit cDC1 via Xcl1, which activates CD8+ T cells via Notch signaling. These processes are controlled by IL-38 acting on γδ T cells (online supplemental figure S4). Taken together, the data presented here suggest a therapeutic potential of anti-IL-38 antibodies for activating antitumor immunity.

## supplementary material

10.1136/jitc-2023-008641 online supplemental file 1

10.1136/jitc-2023-008641 online supplemental file 2

## Data Availability

Data are available upon reasonable request.
