# Supplementary material for: Neutralizing IL-38 activates γδ T cell-dependent antitumor immunity and sensitizes for chemotherapy
Source: J Immunother Cancer. 2024 Aug 28;12(8):e008641. doi: 10.1136/jitc-2023-008641 (PMC11367332; doi:10.1136/jitc-2023-008641)
Supplement: online supplemental file 1 [file jitc-12-8-s001.pdf]

Five Supplementary Figures, Figure Legends, Supplementary Table 2 and 3, and Supplementary Methods

Figure S1

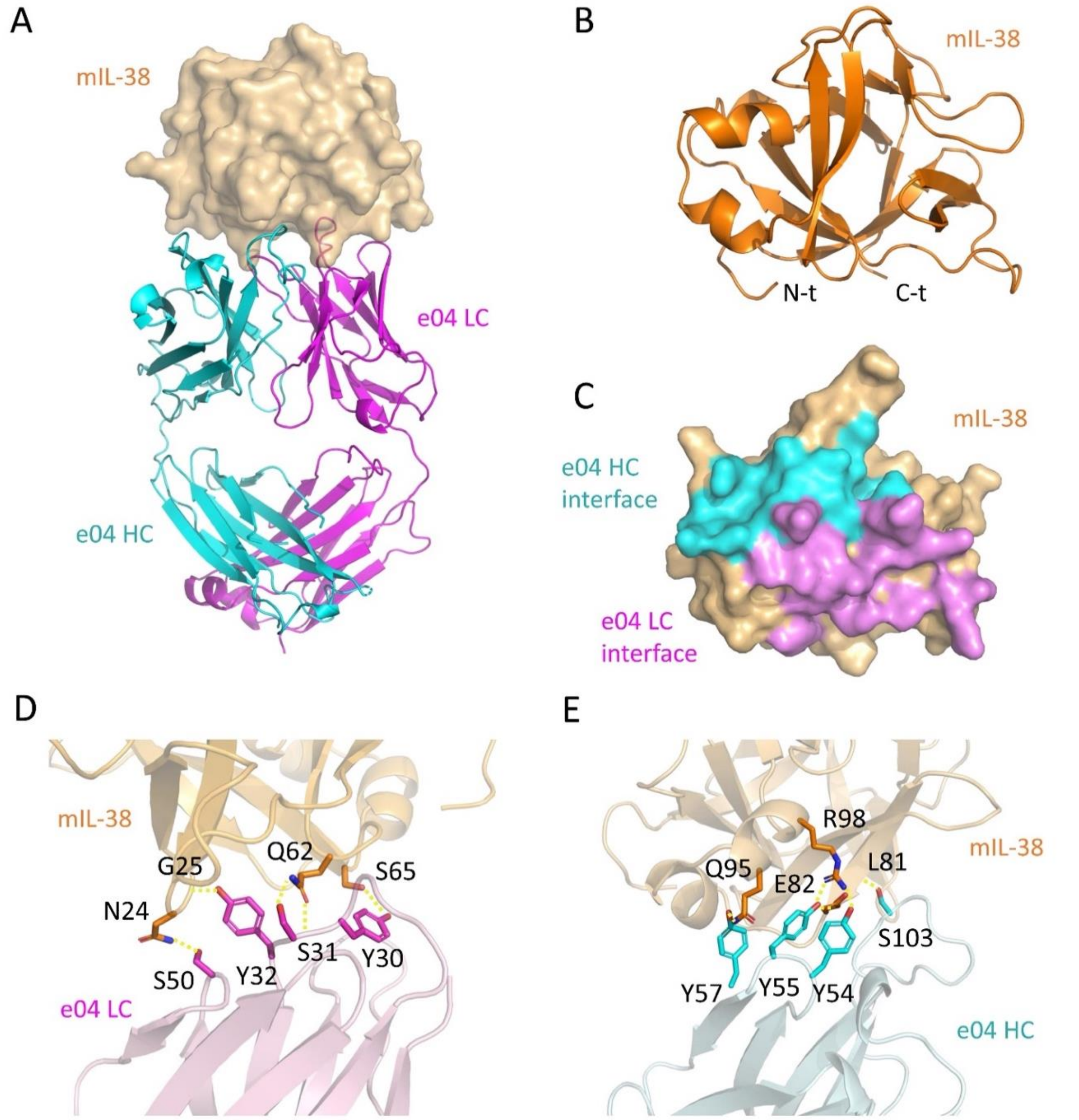

Figure S1. Crystal structure of Fab e04 in complex with mIL-38.
(**A**) The crystal structure of mouse IL-38 (mIL-38) bound to the neutralizing Fab fragment e04 (PDB code, 8Q3J). The heavy (e04 HC, colored in cyan) and light (e04 LC, colored in magenta) chains of the Fab fragment are shown in a ribbon representation. In the complex, the structure of the mIL-38 bound to the Fab e04 is shown in a surface representation. (**B**) Ribbon model of mIL-38 showing its overall structure. N-t and C-t correspond to the N and C termini, respectively. (**C**) Surface representation of mIL-38 showing the mIL-38 epitope recognized by the neutralizing Fab e04 fragment. The complex interfaces for e04 HC and e04 LC are indicated over the mIL-38 surface in cyan and magenta, respectively. (**D,E**) Close-up view of the major contact residues between the Fab e04 and mL-38. Key contact residues between (**D**) e04 LC or (**D**) e04 HC and mIL-38 are shown as sticks and labelled. In (**D,E**), hydrogen bonds are indicated with yellow dashed lines.

Figure S2

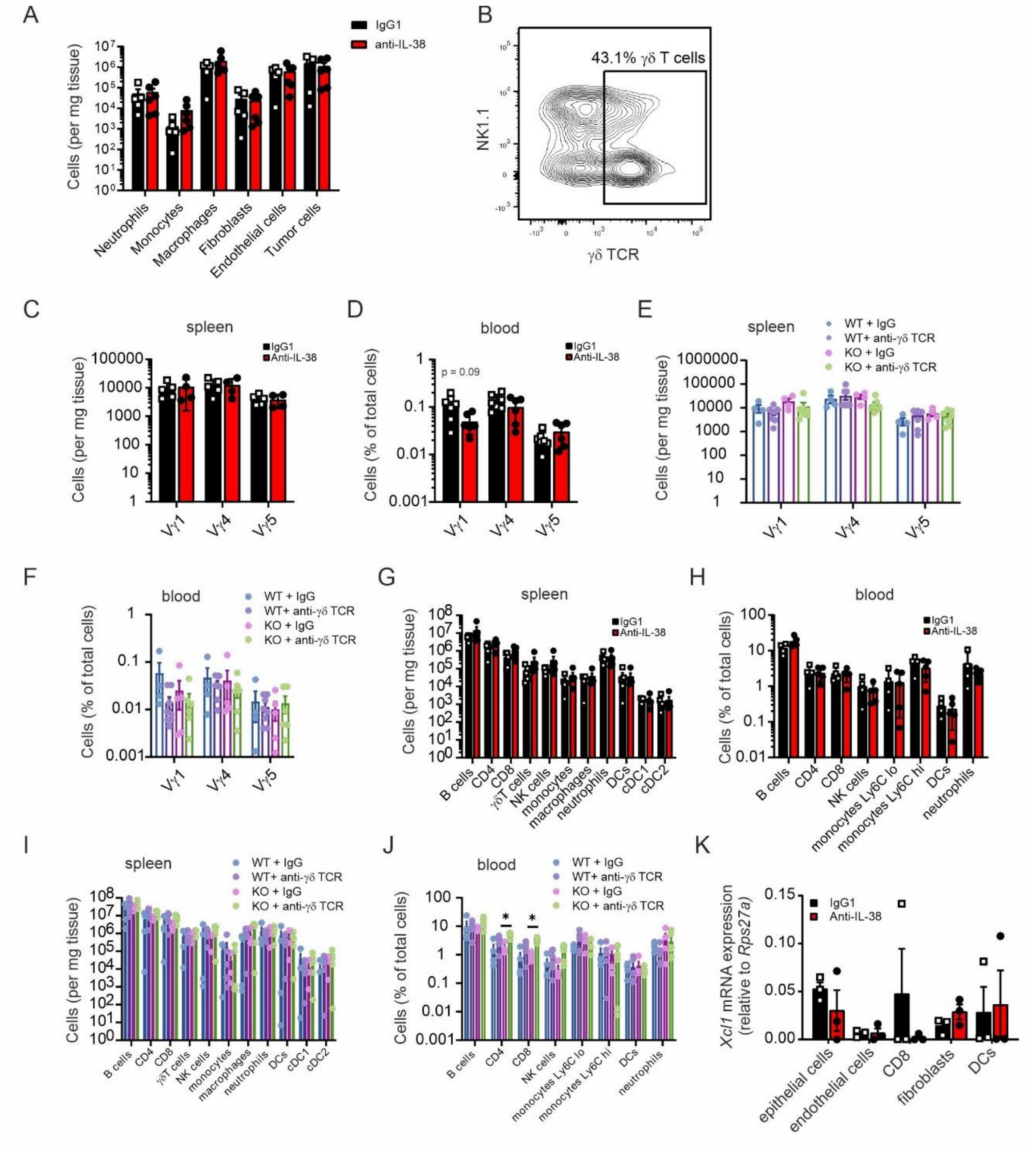

Figure S2. Immune profile of tumors, spleens and blood.

(**A-D,G,H,K**) Polyoma middle T oncogene (PyMT) WT mice were treated with either IgG1 isotype control or anti-IL-38 antibodies (100 μg/mouse) once a week for 5 weeks when the first tumor reached 0.6 cm in diameter. (**A**) Cell composition in tumors was determined by flow cytometry. (**B**) Representative dot plot indicates γδ T cells gating strategy. (**C,D**) The γδ T cell subsets and (**G,H**) immune cell profile in spleen and blood were analyzed by flow cytometry. (**E,F,I,J**) PyMT IL-38 KO and WT mice were treated with either IgG control or γδ-TCR blocking antibodies once mice were 13 weeks old. (**E,F**) The γδ T cell subsets and (**I,J**) immune cell profile in spleen and blood were analyzed by flow cytometry. (**K**) Epithelial cells, endothelial cells, CD8+ T cells, fibroblasts and DCs were isolated by FACS-sorting from PyMT tumors followed by *Xcl1* expression determination by qPCR. Data are shown as means ± SEM. $^*p < 0.05$, $^{**}p < 0.01$, $^{***}p < 0.001$; p-values were calculated using unpaired multiple t-tests with FDR correction.

Figure S3

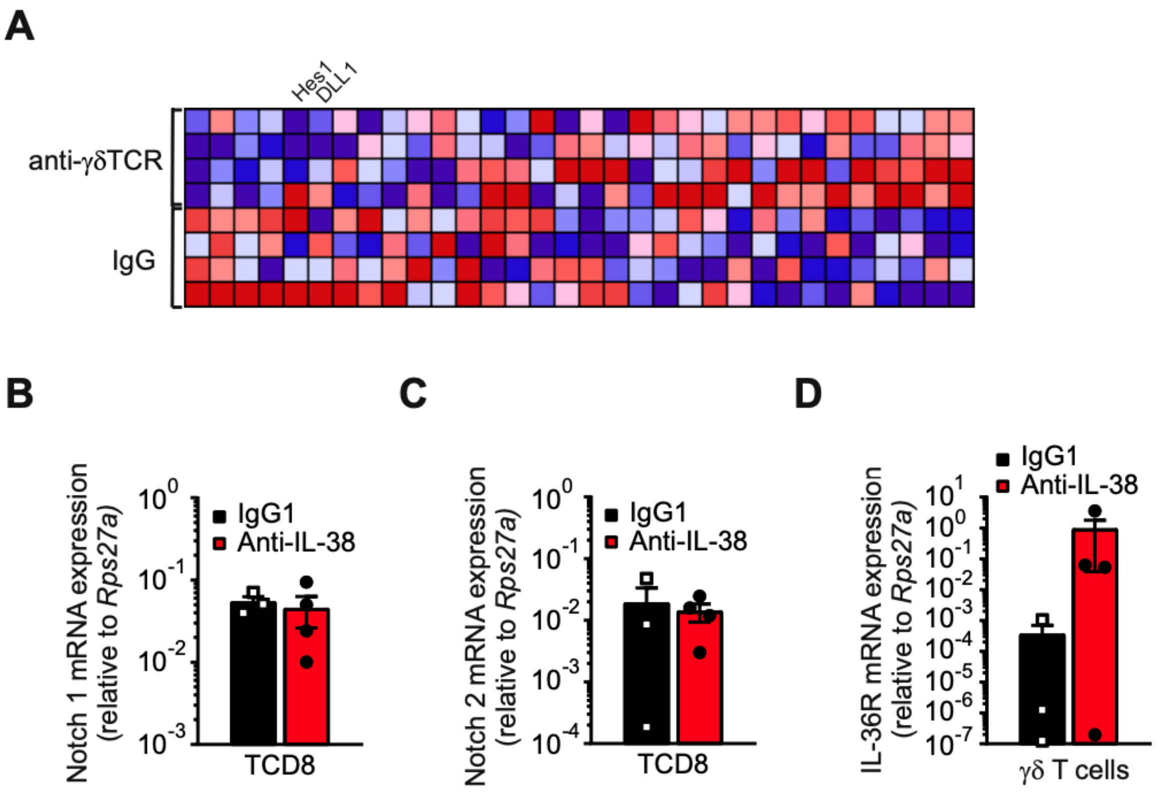

Figure S3. Heatmap and gene expression analysis of Notch pathway genes.
(**A**) PyMT IL-38 KO mice were treated *in vivo* with either IgG isotype control or anti-γδ TCR antibodies. Total RNA was isolated from the tumors and transcriptomes were analyzed by whole transcriptome sequencing. The heatmap indicates the expression profile of the Notch pathway genes from the hallmarks data set in IgG and anti-γδ TCR groups. **(B-D)** CD8+ T cells and γδ T cells were isolated by FACS-sorting from PyMT tumors after *in vivo* IL-38 neutralization, followed by Notch 1, Notch 2 and IL-36R gene expression analysis by qPCR.

Figure S4

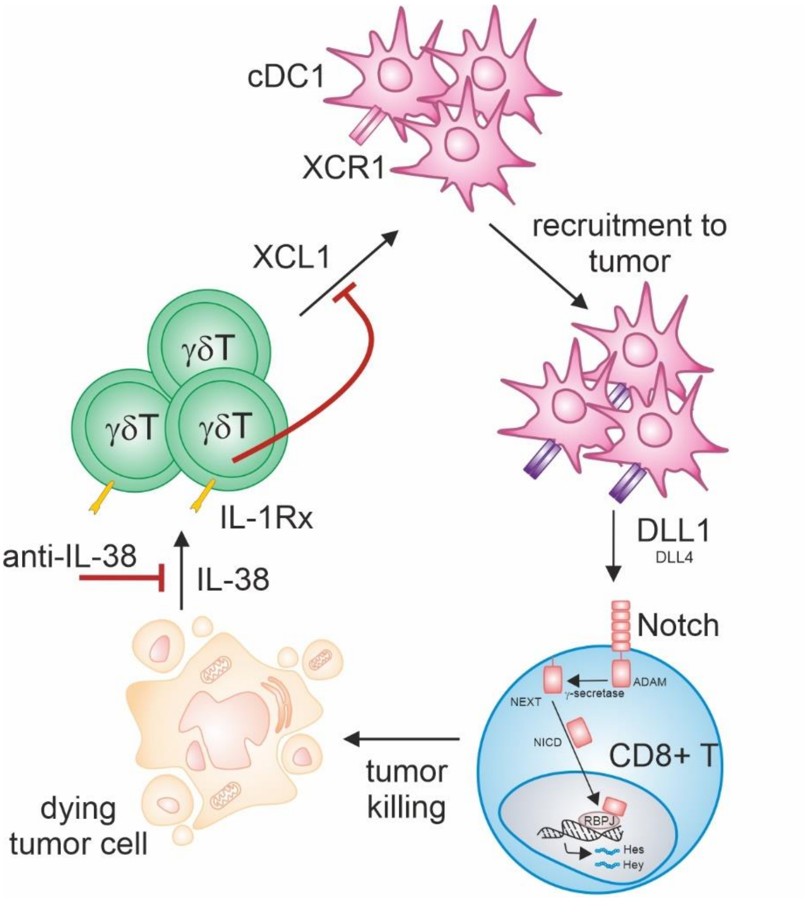

Figure S4. Schematic depiction of the proposed mechanism.
IL-38 neutralization enhances γδ T cells activation leading to cDC1 recruitment to the tumor microenvironment via Xcl1. cDC1 express Dll1 that in turn activates CD8+ T cells via the Notch pathway to increase anti-tumor immunity and hence tumor cell killing.

Figure S5

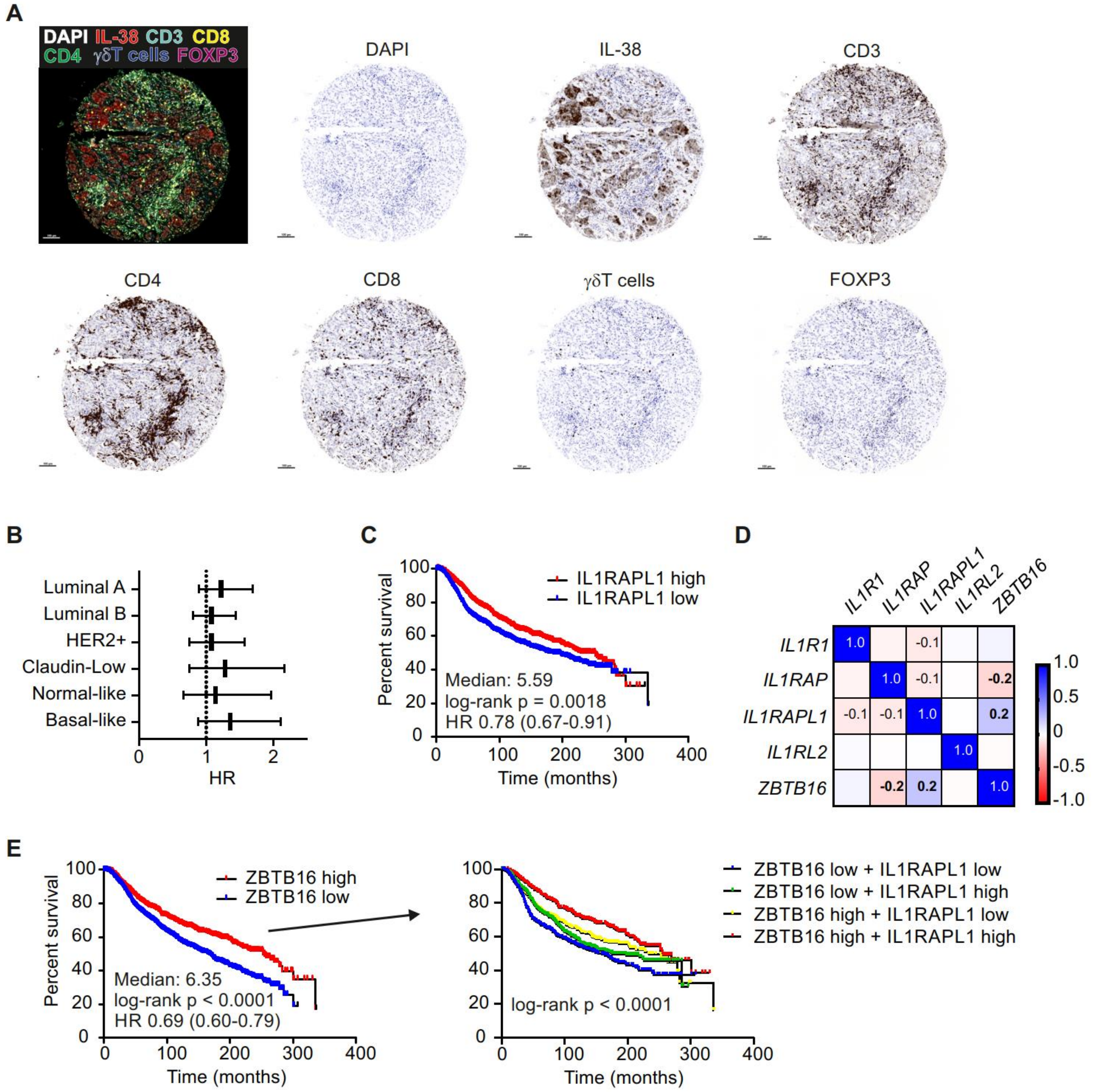

Figure S5. IL-38 pathway in breast cancer patients.
(**A**) Human mammary carcinoma tissue microarrays were analyzed for expression of IL-38 and T cell markers. Representative images of single stains are in one tissue core are displayed. Scale bars are 100 µm. (**B-E**) The METABRIC data set was used to analyze the impact of *IL1RAPL1* on mammary carcinoma patient survival and/or γδ T cell markers. (**B**) Hazard ratios of breast cancer subtypes related to IL-38 (*IL1F10* expression) are shown. (**C**) Survival rates of patients expressing higher (> median) or lower (< median) levels of *IL1RAPL1* are shown. (**D**) Correlation of putative IL-38 receptor expression with expression of *ZBTB16* is shown. Statistically significant difference indicated by bold numbers. (**E**) Survival rates of patients expressing higher (> median) or lower (< median) levels of *ZBTB16*, and of patients expressing higher (> median) levels of *ZBTB16* after further separation into groups expressing higher (> median) or lower (< median) levels of *IL1RAPL1*.

Table S2. Reagents used in the manuscript

REAGENT	SOURCE	CATALOGUE NUMBER/IDENTIFIER
**Antibodies**		
Anti-CD3-PE-CF594	BD Biosciences	562286/RRID:AB_1115330
Anti-CD4-BV711	BD Biosciences	563050/RRID:AB_2737973
Anti-CD140a-PE	BD Biosciences	562776/RRID:AB_2737787
Anti-CD11c-BV711	BD Biosciences	563048/RRID:AB_2734778
Anti-CD326-BV711	BD Biosciences	563134/RRID:AB_2738022
Anti-NK1.1-BV510	BD Biosciences	563096/RRID:AB_2738002
Anti-Ly-6C-PerCP-Cy5.5	BioLegend	128012/RRID:AB_1659241
Anti-CD8-BV650	BioLegend	100742/RRID:AB_2563056
Anti-CD11b-BV605	BioLegend	101257/RRID:AB_2565431
Anti-GITR-FITC	BioLegend	126308/RRID:AB_1089125
Anti-SiglecH-FITC	BioLegend	129604/RRID:AB_1227761
Anti-F4/80-PE-Cy7	BioLegend	123114/RRID:AB_893478
Anti-γδ TCR-APC	BioLegend	118116/RRID:AB_1731813
Anti-Ly-6G-APC-Cy7	BioLegend	127624/RRID:AB_10640819
Anti-HLA-DR (I-A/I-E)	Biolegend	107614/RRID:AB_313329
Anti-CD45- AlexaFluor 700	BioLegend	103128/ RRID:AB_493715
Anti-CD19-APC/Fire 750	BioLegend	115558/RRID:AB_2572120
Anti-CD11c-AlexaFluor 700	BioLegend	117320/RRID:AB_528736
Anti-CD31-PE-Cy7	eBioscience	25-0311-82/RRID:AB_2716949
Anti-MerTK-PE-Cy7	eBioscience	25-5751-82/RRID:AB_2573466
Anti-CD45-VioBlue	Miltenyi	130-118-953/RRID:AB_2751586
Anti-CD90.2-PE	Miltenyi	130-102-489/RRID:AB_2659875
Anti-TCR Vγ1.1-FITC	Biolegend	141104/RRID:AB_10697031
Anti-TCR Vγ2-APC	Biolegend	137708/RRID:AB_10897644
Anti-TCR Vγ3-PE	Biolegend	137504/RRID:AB_10550807
Anti-PD-1-VioBlue	Miltenyi	130-121-437/RRID:AB_2801816
Anti-PD-L1-PE	BioLegend	124307/RRID:AB_2073557
Anti-XCR1-PE	BioLegend	148204/RRID:AB_2563843
Anti-IFNγ-BV421	BD Bioscience	RRID:AB_2738165
Anti-IL-17-PE	Biolegend	RRID:AB_315463
InVivoMab anti-mouse TCR γ/δ	Bio X Cell	BE0070/RRID:AB_1107751
InVivoMab polyclonal Armenian hamster IgG	Bio X Cell	BE0091/RRID:AB_1107773
nVivoMAb human IgG1 isotype control	Bio X Cell	BE0297/RRID:AB_2687817
InVivoPlus anti-mouse CD8α	Bio X Cell	BP0117/AB_10950145
*InVivo*Plus rat IgG2b isotype control, anti-keyhole limpet hemocyanin	Bio X Cell	BP0090/AB_1107780
Anti-CD3	Abcam	ab5690/RRID:AB_305055
Anti-CD8	Cell Signaling	98941/RRID:AB_2756376
Anti-Xcl1	My BioSource	MBS2026530/N/A

Anti-CD4	Cell Signaling	25229/RRID:AB_2798898
Anti-Hes1	Cell Signaling	11988/RRID:AB_2728766
Anti-Rabbit Immunoglobulins/HRP	DAKO	P0448/RRID:AB_2617138
Anti-MHCII	Invitrogen	14-5321-82/RRID:AB_467561
Anti-CD8	Cell Signaling	98941/ RRID:AB_2756376
Anti-F4/80	Cell Signaling	70076/RRID:AB_2799771
Anti-XCL1 antibody	Atlas Antibodies	HPA057725/RRID:AB_2683511
Anti-XCR1 (D2F8T) Rabbit mAb	Cell Signaling	44665/RRID:AB_2799269
Anti-Sheep IgG H&L	Abcam	ab6747/RRID:AB_955453
Anti-rabbit HRP	Akoya Biosciences	ARR1001KT/N/A
Histofine Simple Stain Mouse MAX PO (Rat) Anti-Rat	Histofine	414311F/N/A
Normal Goat IgG Control	R&D Systems	AB-108-C/RRID:AB_354267
Mouse XCL1/Lymphotactin Antibody	R&D Systems	AF486/RRID:AB_2216915
Anti-Pan-Cytokeratin	Abcam	ab27988/RRID:AB_777047
Anti-IL-38 human	ThermoFisher	14-7385-82/RRID:AB_2572906
Anti-CD3 human	Ventana	790-434/RRID:AB_2335978
Anti-δ TCR human	Santa Cruz	sc-100289/RRID:AB_1130061
Anti-CD8 human	DAKO	M7103/RRID:AB_2075537
Anti-CD4 human	Abcam	ab133616/RRID:AB_2750883
Anti-FOXP3 human	Abcam	ab20034/RRID:AB_445284
**Chemical, Peptides, and Recombinant proteins**		
Fc Blocking reagent	Miltenyi Biotec	130-092-575
Flow cytometry absolute count standard	Bangs laboratories	580
Brilliant Staining buffer	BD Biosciences	563794
Brefeldin A	Sigma Aldrich	B6542
Protein Transport Inhibitor	BD Bioscience	554724
Cell Stimulation Cocktail	eBioscience	00-4970-93
GlutaMax	ThermoFisher	35050038
Doxorubicin-hydrochlorid	Teva Pharma	74236.00.00
rm IL-23	Bio-Techne	1887 ML
rmIL-1β	Bio-Techne	401-ML
rm-IL-38	Adipogen	AG-40B-0101-C010
rm IL-2	Immuno Tools	12340024
DAPT, γ-secretase inhibitor	Abcam	ab120633
**Critical Commercial Assays**		
Absolutely RNA Microprep Kit	Agilent	400805
SensiScript RT Kit	Qiagen	205211
Maxima First Strand cDNA Synthesis Kit for RT-qPCR	Thermo Fisher Scientific	K1641
Easysep Mouse Pan-DC Enrichment Kit II	StemCell Technologies	19863

Mouse Xcl1/Lymphotactin DuoSet ELISA Kit	R&D Systems	DY486
Mouse IL-17A Flex Set	BD Bioscience	560383
BD Cytofix/Cytoperm	BD Bioscinece	554714
Mouse IFN-γ Flex Set	BD Bioscience	558296
RNAScope® Multiplex Fluorescent V2 kit	Advanced Cell Diagnostics	323110
Opal 7-Color Fluorescent IHC Kit	Perkin-Elmer	NEL797001KT
Tumor dissociation Kit mouse	Miltenyi Biotec	130-096-730
Qubit dsDNA HS and BR Assay Kit	Thermo Fisher Scientific	Q32854
Qubit RNA High Sensitivity (HS)	Thermo Fisher Scientific	Q32852
QuantSeq 3' mRNA-Seq Library Prep Kit FWD with 12 nt Unique Dual Indices	Lexogen	114
High Sensitivity D1000 Screen Tape	Agilent	5067-5584
RNA Screen Tape	Agilent	5067-5576
NextSeq 1000/2000 P2 Reagents (100 Cycles)	Ilumina	20046811
RNA Clean & Concentrator-5	Zymo Research	R1015
**Oligonucleotides**		
Probe to Mm-IL1F10	Advanced Cell Diagnostics	524771
**Software and Agorithms**		
FlowJo V10	Tree Star	https://www.flowjo.com/
QuantStudio 3 and 5 Real-Time PCR System Software	Thermo Fisher Scientific	https://www.thermofisher.com/de/de/home/global/forms/life-science/quantstudio-3-5-software.html
Graph Pad Prism 9	GraphPad Software	https://www.graphpad.com/
InForm V2.6 Software	PerkinElmer	https://www.akoyabio.com/phenoimager/software/inform-tissue-analysis-software/
BlueBee Genomics	Lexogen	https://faqs.lexogen.com/faq/bluebee-genomics-pipelines
GenePattern	Broad Institute	https://www.genepattern.org/#gsc.tab=0

Table S3. Data collection and refinement statistics

**Data collection statistics**	
Beamline	DESY P14
Wavelength (Å)	0.9763
Space Group	$P\ 1\ 2_1\ 1$
Cell dimensions (Å)	a = 73.80 b = 66.94 c = 129.05<br>α = 90.00 β = 104.73 γ = 90.00
Resolution (Å)	70.11-2.35
Unique reflections	50677
Redundancy	6.4
Completeness (%)	99.2
**Refinement statistics**	
Reflections in test set	4085
R_{cryst}	23.3
R_{free}	28.2
**Number of groups**	
Protein residues	1148
Ions and ligand atoms	4
Water	109
Wilson B-factor	54.1
**RMSD from ideal geometry**	
Bond length (Å)	0.009
Bond angles (°)	1.13
**Ramachandran Plot Statistics**	
In Favoured Regions (%)	95.28
In Allowed Regions (%)	4.18
Outliers (%)	0.53
PDB accession code	8Q3J

Methods

Spleen and blood single cell preparation

Spleens were mechanically dissociated in phosphate-buffered saline (PBS) containing 1mM EDTA and blood was collected with tubes containing EDTA K to avoid coagulation followed by erythrocyte lysate with RB Lysis Buffer (BD Bioscience).

Mouse IL-38 production

The design of mIL-38 and the general purification strategy was done as described before (1, 2). For structural studies, mIL-38 was expressed as a Ub-tagged fusion protein using an *E. coli* BL21 strain (New England Biolabs, C2530H). Ub-mIL-38 expression was induced with 0.25 mM isopropyl β-D-1-thiogalactopyranoside (IPTG) at a cell density of OD_{600} = 0.5. After incubation for 16 h at 18 °C and 250 rpm, the cells were harvested by centrifugation for 30 min at 4000 × g. Then, the cells were lysed by sonication in Lysis buffer (PBS buffer supplemented with 300 mM NaCl and 1 mM β-mercaptoethanol, pH 7.5). After centrifugation at 20,000 × g for 30 min at 4 °C, the supernatant was filtered through a 0.45 µm filter. The Ub-mIL-38 fusion protein was then purified using a HisTrapTM FF Ni-column (Cytiva Life Sciences) equilibrated with the Lysis buffer. The bound protein was eluted by applying an imidazole gradient starting from 0 to 100% of the Elution buffer (PBS buffer, pH 7.5, supplemented with 300 mM NaCl and 1 mM β- mercaptoethanol and 200 mM Imidazol). All the eluted fractions containing Ub-mIL-38 were pooled and purified in a HiLoad^{TM} 16/600 75 pg column (Cytiva Life Sciences) equilibrated with a PBS with 300 mM NaCl, pH 7.5 buffer. The resultant purified Ub-mIL-38 fusion protein was cleaved with 5 µM His_6-USP2 protease produced *in house.* Afterwards, the mIL-38 protein was separated from the cleaved Ub tag on a second size exclusion chromatography step. To improve

protein stability, the final purified mIL-38 was dialyzed against 20 mM citrate, 300 mM NaCl, pH 5.0 buffer.

Large-scale production of Fab e04 for structural studies

For structural studies, the fab e04 light and heavy chains were subcloned into the pTriEx-6 (Merk Millipore) and pTriEx-7 (Merk Millipore) expression vectors, respectively. Fab e04 fragment was produced extracellularly by transient transfection of Expi293F suspension cells, similar as described for other Fab fragments and structurally complex secreted proteins (3-6). Briefly, 500 µg of DNA of each construct were mixed with polyethyleneimine (PEI) (linear 25 kDa, Polysciences Inc.) at a final ratio of 1:3 DNA:PEI (w:w) in 50 ml of fresh FreeStyle 293 Expression Medium (Thermo Scientific). DNA-PEI complexes were then added to the culture at a cell density of $1x10^6$ cells/ml and incubated in 500 ml flasks on a rotary shaker (120 rpm) at 37°C, 8% CO_2 and 70% humidity. Following transient co-expression in Expi293F cells for 7 days, secreted Fab e04 fragment was purified by using a TALON Metal Affinity Resin (Takara Bio) equilibrated with PBS buffer, pH 7.4 with 500 mM NaCl. After washing with 5 column volumes of the same buffer, the bound protein was eluted with PBS buffer, pH 7.4, containing 500 mM NaCl and 150 mM imidazole. Finally, the eluted fractions with the purified Fab eo4 fragment were concentrated and loaded into a PD-10 desalting column SephadexTM G-25 M to exchange the buffer to PBS, pH 7.4 with 500 mM NaCl.

Fab e04-mIL-38 complex formation and crystallization experiments

To obtain the Fab e04-mIL-38 complex, the purified Fab e04 fragment were mixed with mIL-38 in a 1:1.2 (Fab e04:mIL-38) molar ratio. After incubation for 2 h at 4 ºC, the complex was purified using a size-exclusion chromatography (Superdex 75 10/300 GL)

using a PBS-citrate buffer, pH 6.1 with 430 mM NaCl. For crystallization experiments, the Fab e04-mIL-38 complex was concentrated until 15 mg/ml using a 30K Amicon (Merck-Millipore). At the same time, the buffer was exchanged to 4 mM Phosphate-citrate buffer, pH 6.1 with 135 mM NaCl. Monoclinic crystals formed by the Fab e04-mIL-38 complex were grown at 18 °C using the sitting-drop vapor-diffusion method. The reservoir solution contained 100 mM SPG buffer, pH 8.7 and 27.5% (w/v) polyethylene glycol 1500 (PEG1500). Single crystals appeared after 15 days in 0.5 ul drops mixed from equal volumes of protein solution and reservoir solution. The crystals were recovered and cryoprotected in reservoir buffer containing 20% Ethylene glycol and flash-frozen in liquid nitrogen prior to diffraction analysis.

X-ray data collection and Fab e04-mIL-38 complex structure determination

Diffraction for the Fab e04 in complex with mIL-38 was recorded from cryo-cooled crystals (100K) at the p14 beamline operated by EMBL Hamburg at the PETRA III storage ring from the DESY synchrotron (Hamburg, Germany). Diffraction data was integrated and merged using XDS (7) and scaled and reduced using CCP4 software (8).

The structure of the Fab e04 in complex with mIL-38 was determined from the X-ray data by molecular replacement using previously solved structures of a Fab fragment (PDB accession code 6O3A) (9) and the x-ray crystal structure of human IL-38 (PDB accession code 5BOW) as a reference models. Manual model building of the Fab e04-mIL-38 complex, and refinement were carried out using Coot (version 0.9.8.8) (10) and phenix.refine from Phenix (11). Table S3 provides a summary of the refined model parameters. The atomic coordinates of Fab e04 in complex with mIL-38 have been deposited in the Protein Data Bank (Accession No. 8Q3J). Structural representations

were prepared with PyMOL (Schrödinger, L., and W. DeLano. 2020. PyMOL. available at: http://www.pymol.org/pymol.)

References

1. Han Y, Mora J, Huard A, da Silva P, Wiechmann S, Putyrski M, et al. IL-38 Ameliorates Skin Inflammation and Limits IL-17 Production from gammadelta T Cells. Cell Rep. 2019;27(3):835-46 e5.
2. Mora J, Schlemmer A, Wittig I, Richter F, Putyrski M, Frank AC, et al. Interleukin-38 is released from apoptotic cells to limit inflammatory macrophage responses. J Mol Cell Biol. 2016;8(5):426-38.
3. V J, K B, A W, S W, M H, A F, T S. High level transient production of recombinant antibodies and antibody fusion proteins in HEK293 cells. BMC biotechnology. 2013;13.
4. MC G-G, J G-P, E B, R F-A, GB B, PJ L, et al. Crystal structure and mechanism of human carboxypeptidase O: Insights into its specific activity for acidic residues. Proceedings of the National Academy of Sciences of the United States of America. 2018;115(17).
5. J G-P, S T, MC G-G, S D, FX A, J L, LD F. Substrate Specificity and Structural Modeling of Human Carboxypeptidase Z: A Unique Protease with a Frizzled-Like Domain. International journal of molecular sciences. 2020;21(22).
6. J G-P, S M, FX A, S T, J L. Enhanced Production of ECM Proteins for Pharmaceutical Applications Using Mammalian Cells and Sodium Heparin Supplementation. Pharmaceutics. 2022;14(10).
7. W K. XDS. Acta crystallographica Section D, Biological crystallography. 2010;66(Pt 2).
8. MD W, CC B, KD C, EJ D, P E, PR E, et al. Overview of the CCP4 suite and current developments. Acta crystallographica Section D, Biological crystallography. 2011;67(Pt 4).
9. S R, M B, M T, K L, F L, Y J, et al. Structure-guided design fine-tunes pharmacokinetics, tolerability, and antitumor profile of multispecific frizzled antibodies. Proceedings of the National Academy of Sciences of the United States of America. 2019;116(14).
10. P E, B L, WG S, K C. Features and development of Coot. Acta crystallographica Section D, Biological crystallography. 2010;66(Pt 4).
11. D L, PV A, ML B, G B, VB C, TI C, et al. Macromolecular structure determination using X-rays, neutrons and electrons: recent developments in Phenix. Acta crystallographica Section D, Structural biology. 2019;75(Pt 10).
